# C9orf72 deficiency impairs the autophagic response to aggregated TDP-25 and exacerbates TDP-25-mediated neurodegeneration in vivo

**DOI:** 10.1186/s40478-025-02061-5

**Published:** 2025-06-28

**Authors:** Lilian Tsai-Wei Lin, Marc Shenouda, Philip McGoldrick, Agnes Lau, Janice Robertson

**Affiliations:** 1 Tanz Centre for Research in Neurodegenerative Diseases, University of Toronto, Krembil Discovery Tower, 60 Leonard Ave., Toronto, ON M5T 0S8 Canada; 2 Department of Laboratory Medicine and Pathobiology, University of Toronto, Toronto, ON M5S 1A1 Canada

**Keywords:** Neurodegeneration, Amyotrophic lateral sclerosis, Frontotemporal dementia, TAR DNA-binding protein 43, C9orf72, Protein aggregation, Autophagy

## Abstract

Cytoplasmic aggregates of the predominantly nuclear TAR DNA-binding protein 43 (TDP-43) are a pathological hallmark of amyotrophic lateral sclerosis (ALS) and frontotemporal dementia (FTD) cases caused by G_4_C_2_ hexanucleotide repeat expansions in *C9orf72* (C9-ALS/FTD). While these repeat expansions are associated with both gain- and loss-of-function mechanisms, the contribution of C9orf72 loss of function to disease pathogenesis remains unclear. C9orf72 has been shown to regulate autophagy, and its deficiency has been shown to exacerbate phenotypes in gain-of-function G_4_C_2_ models, implicating impaired autophagic clearance in disease pathogenesis. Here, we directly test whether C9orf72 deficiency exacerbates TDP-43 pathology and neurodegeneration in vivo. Using AAV9-vectors to drive neuron-specific expression of pathologically relevant C-terminal species of TDP-43, TDP-35 and TDP-25, we established models of TDP-43 pathology that recapitulate key disease features, including cytoplasmic aggregates, motor and cognitive decline, and neuronal loss. TDP-25 expression in particular produced robust, abnormally phosphorylated, ubiquitinated and p62-labelled cytoplasmic aggregates, modelling TDP-43 pathology in disease. Loss of C9orf72 in TDP-25-expressing mice accelerated the onset of motor deficits, increased neurodegeneration, and impaired the autophagic response to TDP-25 expression. These findings reveal that C9orf72 deficiency disrupts autophagy and exacerbates TDP-25-mediated toxicity in vivo, supporting a contributory role for C9orf72 loss-of-function in driving neurodegeneration in C9-ALS/FTD.

**Supplementary Information:**

The online version contains supplementary material available at 10.1186/s40478-025-02061-5.

## Introduction

Amyotrophic lateral sclerosis (ALS) is an adult-onset neurodegenerative disease characterized by loss of motor neurons in the brain and spinal cord, leading to progressive paralysis and death, usually within 2–5 years of diagnosis [1]. Cognitive and behavioural impairments are observed in approximately 50% of ALS patients, with 10–15% meeting the clinical criteria for frontotemporal dementia (FTD), placing ALS and FTD on a disease spectrum unified by overlapping genetic, pathological and molecular features [2]. The pathological hallmark of ALS/FTD is the cytoplasmic mislocalization and aggregation of TAR DNA-binding protein 43 (TDP-43), a predominantly nuclear DNA/RNA binding protein, in neurons and glia [3, 4]. Cytoplasmic TDP-43 aggregates are abnormally phosphorylated, ubiquitinated, and co-labelled with p62, implicating dysfunction of both the ubiquitin-proteasome system (UPS) and autophagic degradation pathways [5]. Consistent with this, several genes involved in protein homeostasis pathways are causative of ALS/FTD, including *SQSTM1*, *OPTN*, *TBK1*, *VCP*, *UBQLN2*, *GRN* and *C9orf72* [6]. Among these, hexanucleotide (G_4_C_2_) repeat expansions in *C9orf72* are the most common genetic cause of ALS/FTD (C9-ALS/FTD). These expansions are thought to contribute to disease through three non-mutually exclusive mechanisms: *C9orf72* haploinsufficiency, RNA foci-mediated toxicity, and toxicity from aberrantly translated dipeptide repeat proteins (DPRs) [7–9]. Although none of these mechanisms has been definitively established as the primary driver of pathogenesis, it is generally acknowledged that they may act synergistically to promote neurodegeneration. Multiple studies, including our previous work, have reported downregulation of *C9orf72* transcription and reduced C9orf72 protein levels in C9-ALS/FTD patient tissues [7, 8, 10–13], pointing to a potential role for haploinsufficiency in disease pathogenesis. C9orf72 has demonstrated roles in autophagy regulation, forming a complex with SMCR8 and WDR41 to promote autophagy initiation through ULK1 [14, 15] and to negatively regulate autophagy through an interaction with mTOR [16, 17]. Loss of C9orf72 has been shown to disrupt autophagy and exacerbate DPR toxicity across multiple models [18–22]. This suggests that autophagic dysfunction caused by loss of C9orf72 could lead to impaired clearance of TDP-43 aggregates and contribute to neurodegeneration in C9-ALS/FTD.

To determine whether C9orf72 loss of function contributes to TDP-43-mediated neurodegeneration, we generated two in vivo models using AAV9-mediated expression of pathologically relevant C-terminal species of TDP-43, TDP-35 and TDP-25, in mouse brain. Both models exhibited region-specific transgene expression, behavioural deficits and neuronal loss. Neurotoxicity in TDP-25-expressing mice was associated with the formation of neuronal cytoplasmic TDP-25 aggregates that were abnormally phosphorylated, ubiquitinated, and p62-positive, modelling TDP-43 pathology in disease. We found that loss of C9orf72 exacerbated phenotypes in TDP-25-expressing mice, accelerating the onset of motor dysfunction and increasing neuronal loss. Importantly, loss of C9orf72 impaired the autophagic response to TDP-25 expression. These findings support that C9orf72 deficiency contributes to TDP-43-mediated neurodegeneration through impaired autophagy.

## Materials and methods

### Animals

All animal work was conducted in accordance with the Canadian Council on Animal Care guidelines and approved by the University of Toronto Animal Care Committee. C57BL/6 mice were purchased from Charles River. *C9orf72* knockout mice were a generous gift from Dr. Don Cleveland (UCSD) and Dr. Clothilde Lagier-Tourenne (Harvard University) and genotyped as previously described [23].

### AAV9 production and preparation

rAAV9 viral vectors expressing EGFP, EGFP TDP-35, and EGFP-TDP-25 under control of the human synapsin promoter (hSYN1) and human growth hormone (hGH) first intron enhancer were produced by Virovek (CA, USA) as previously described [24, 25]. AAV2 inverted terminal repeat (ITR) sequence was used to package the virus, and Woodchuck Hepatitis Virus (WHV) Posttranscriptional Regulatory Element (WPRE) and SV40 early polyadenylation signals (SV40pA) were added to stabilize and enhance gene expression. Prior to neonatal injections, the virus was diluted to ~ 2 × 10^12^ vg/mL in sterile Phosphate Buffered Saline (PBS, Gibco) and FastGreen dye (Sigma-Aldrich) was added to a final concentration of 0.01% as previously described [24].

### Neonatal i.c.v. Injections

Intracerebroventricular (i.c.v.) injections in neonatal mice (P0) were approved by the University of Toronto Animal Care Committee. Injections were performed as previously described [24]. Cryoanesthetized pups were positioned on a lit transparent platform to visualize the transverse sinus. Using a 33-gauge, 30° beveled needle (point style 4) and gastight syringe retrofit with a Neuros adaptor and blind stop (Hamilton), 7 µL of viral vector was injected into the right lateral ventricle (2 mm rostral and 1 mm lateral to the superior sagittal sinus, 2 mm depth). FastGreen dye spread was monitored for injection efficiency. Pups were observed post-injection and returned to home cages.

### Grip strength

Grip strength was assessed using the Bioseb Grip Strength Test (BIO-GS3) per the manufacturer’s protocol. Mice grasped a metal grid with their forelimbs while hind limbs remained suspended. Mice were then pulled horizontally by the tail and peak tension before grip loss was recorded in grams. Mice underwent training for two days, followed by a rest day before testing. Four trials were conducted on both training and test days, with results averaged. Mouse weights were recorded on test day.

### Novel object recognition

Memory index (MI) was assessed using the novel object recognition (NOR) task with a 1-hour delay. Mice were habituated to the testing area for 10 min daily over four days. On test day, mice explored two objects for 10 min (phase 1). After one hour, one familiar and one novel object were presented for 5 min (phase 2). Object interaction frequency and duration were recorded using ODLog (Macropod). The experimenter is blinded to cage numbers. Interaction was defined as any time the nose of the mice came within 1 cm of the object with the nose pointing towards the object. The MI was then calculated by dividing the difference in interaction time between the novel object and the familiar object by the sum of the total time spent interacting with the two objects [MI= (time novel - time familiar)/(time novel + time familiar)].

### Fear conditioning

Mice underwent cued and contextual fear conditioning in Freeze Monitor System chambers (San Diego Instruments) for three days. On day 1, mice were placed in Context A (white walls, metal grid floor, ethanol smell) for 180s. A tone sounded at 60s and lasted for 90s, with 2s, 0.6 mA foot shocks at 88s and 148s. On day 2, mice explored Context A for 300s without the tone; contextual memory was assessed via freezing behavior, recorded by laser beam breaks and analyzed with Freeze Monitor software. On day 3, mice explored Context B (brown walls, plastic floor, water smell) for 300s. The tone played at 120s for 180s, and cued memory was measured via freezing behavior. Contextual and cued fear memory were calculated as percent time freezing on days 2 and 3, respectively.

### Open field

Mice were placed in the center of a black box (100 cm x 100 cm) for 10 min. Their movement during this time period was tracked through the ANY-Maze video tracking software (ANY-Maze). The software then calculated the total distance traveled, the amount of time spent in the center, and the mean speed of the mice.

### Immunofluorescence for FFPE sections

Mice were anesthetized by intraperitoneal injection (i.p.) of ketamine/xylazine (100/10 mg/kg) and transcardially perfused with ice-cold PBS followed by 10% formalin (Sigma-Aldrich). Brains were post-fixed in formalin for 7 days at 4 °C, transferred to 70% ethanol for another 7 days, then stored in 70% ethanol. Samples were paraffin-embedded, sectioned (6 μm), and mounted on slides. The formalin fixed-paraffin embedded (FFPE) brain sections on slides were heated at 60 °C for 20 min, deparaffinized in xylene (Sigma-Aldrich) and rehydrated in a series of solutions (1:1 xylene: ethanol, 100% ethanol, 95% ethanol, 75% ethanol, 50% ethanol, MiliQ water) for 5 min each. Heat-induced epitope retrieval (HIER) was performed in Tris-EDTA, pH 9.0 (TE9) for 15 min using a pressure cooker. Brain sections on slides were cooled on the benchtop and rinsed under running water for 5 min, then washed with Tris-buffered saline (TBS) containing 0.1% Tween-20 (TBST) twice for 5 min each. Tissues were blocked and permeabilized in 10% donkey serum (Gibco), 3% bovine serum albumin (Sigma-Aldrich), and 0.4% Triton X-100 (Sigma-Aldrich) blocking buffer for 1 h at room temperature. Tissues were stained with primary antibody diluted in blocking buffer [anti-GFP (Aves Labs, 1:1000); anti-NeuN (Millipore, 1:500); anti-TDP-43 (Proteintech, 1:500); anti-Ubiquitin (Millipore, 1:500); anti-C-terminal TDP-43 (Sigma-Aldrich, 1:500); anti-N-terminal TDP-43 (Aviva, 1:500); anti-phospho-TDP-43 (Ser409/410) (BioLegend/Millipore, 1:500); anti-p62 (Abcam, 1:500); anti-GFAP (Millipore, 1:500); anti-Iba1 (Abcam, 1:1000); anti-LC3A/B (Abcam, 1:1000)] overnight at 4^o^C. Tissue slides were then washed with TBST and incubated with Alexa Fluor secondary antibodies (Life Technologies, 1:500) and DAPI stain (Thermo Fisher, 1:10,000) diluted in blocking buffer for 2 h at room temperature. Finally, the tissue was washed in TBST, and coverslips were mounted using ProLong Gold Antifade Mountant with DAPI (Invitrogen). Images were taken with a Zeiss LSM880 with Airyscan confocal microscope and Zen Black software (Zeiss).

### Immunofluorescence for free-floating sections

Mice were perfused as above and post-fixed in 4% paraformaldehyde (PFA) for 7 days at 4 °C followed by 30% sucrose in PBS, pH 7.4 for 7 more days. Serial brain sections were cut at 40 μm thickness on a freezing sledge microtome (Thermo Fisher), and the tissue sections stored in antifreeze (30% glycerol, 30% ethoxyethanol, 40% PBS) at -20 °C, as previously described. Free-floating sections were washed in PBS, permeabilized using 1X PBS with 0.4% Triton X-100 (PBST), then incubated in blocking buffer [10% donkey serum (Gibco), 3% bovine serum albumin (Sigma-Aldrich), and 0.4% Triton X-100 (Sigma-Aldrich)] for 1 h at room temperature. Tissue sections were then incubated with primary antibodies diluted in blocking buffer [anti-GFP (Aves Labs, 1:1000); anti-NeuN (Millipore, 1:500); anti-phospho-TDP-43 (Ser409/410) (BioLegend/Millipore, 1:500); anti-p62 (Abcam, 1:500)] overnight at 4 °C. Sections were washed in PBS and incubated with Alexa Fluor secondary antibodies (Life Technologies, 1:500) and DAPI stain (Thermo Fisher, 1:10,000) diluted in blocking buffer for 2 h at room temperature. Sections were then mounted onto Superfrost plus slides (Fisherbrand) and coverslipped with ProLong Gold Antifade Mountant with DAPI (Invitrogen). Images were taken using a Leica DMI6000B Inverted microscope or Leica DM6000B upright microscope with a Hamamatsu Orca-ER digital camera and Volocity Acquisition Suite (v6.3, Perkin Elmer).

### Stereology

Free-floating mouse brain sections (~ 1 mm lateral) were analyzed for viral-induced expression of EGFP, EGFP-TDP-35, or EGFP-TDP-25. Sections were collected every 200 μm, stained, and examined for expression and regional aggregation of TDP-25. Sagittal sections (~ 1 mm lateral, motor cortex; ~3 mm lateral, entorhinal cortex) were stained [anti-GFP (Aves Labs, 1:1000); anti-NeuN (Millipore, 1:500)] to quantify neuronal density. Coronal sections (Bregma ~-3.00 mm, entorhinal cortex; ~1.10 mm, motor cortex) were stained [anti-GFP, anti-NeuN, anti-p62 (Abcam, 1:500)] to quantify neuronal density, TDP-25 aggregation, and p62 puncta. Quantification was performed using CellProfiler (v3.1.9) and ImageJ. For EGFP, EGFP-TDP-35, and EGFP-TDP-25 sagittal sections, 10X images of the region of interest (*n* = 3, > 2000 cells per animal) were used for neuronal density. Coronal sections quantified TDP-25 aggregates in the entorhinal cortex, with two 10X non-overlapping images per hemisphere (*n* > 3 per cohort, > 5000 cells per animal). p62 puncta were quantified in 20X images (*n* = 3 per cohort, 260–600 GFP-positive cells per animal). For motor cortex analysis, one 10X image was taken per hemisphere above the arch of the cingulate gyrus (*n* = 3 per cohort, > 4800 cells per animal). Results from both hemispheres were averaged per animal. Neurons were defined as DAPI- and NeuN-positive, and GFP-positive cells were DAPI-, NeuN-, and GFP-positive. Aggregates in the 90th percentile and above were classified as ‘large’, and those in the 10th percentile and lower as ‘small’.

### Cell culture

Parental and C9KO HeLa cell lines were described previously [24]. Cells were cultured in Dulbecco’s Modified Eagle Medium (DMEM; Gibco) with 10% fetal bovine serum (FBS; Gibco) and 1% penicillin-streptomycin (Gibco) at 37^o^C and 5% CO_2_. Cells were plated on poly-D-lysine-coated glass coverslips in 24-well plates, reaching 70–80% confluency before transient transfection with 1 µg pEGFP or pEGFP-TDP-25 using Lipofectamine LTX with PLUS Reagent (Invitrogen) in Opti-MEM (Gibco). After 3 h, Opti-MEM was replaced with DMEM. For C9orf72 (C9) rescue, 0.5 µg pcDNA3 or FLAG-C9 DNA was co-transfected with 0.5 µg pEGFP-TDP-25. Autophagy modifiers bafilomycin (100 nM) and rapamycin (10 µM) were added 24 h post-transfection for 4 h. DMSO-treated cells served as controls.

### Immunofluorescence of HeLa cells and quantification of aggregates

Coverslips with cells were washed in PBS (pH 7.4) and fixed in 4% PFA for 15 min at room temperature 24 h post-transfection (28 h for autophagy-modifier-treated cells). After fixation, cells were washed in PBS and blocked for 30 min in buffer [10% donkey serum (Gibco), 3% BSA (Sigma-Aldrich), 0.4% Triton X-100 (Sigma-Aldrich)]. Cells were then incubated with primary antibodies [anti-GFP (Aves Labs, 1:2000); anti-p62 (Abcam, 1:1000)] for 1 h at room temperature, followed by PBS washes and a 30-min incubation with Alexa Fluor secondary antibodies (Life Technologies, 1:1000) and DAPI (Thermo Fisher, 1:10,000) in blocking buffer. Coverslips were washed, mounted on Superfrost Plus slides (Fisherbrand) with ProLong Gold Antifade Mountant (Invitrogen), and imaged using a Leica DMI6000B Inverted microscope with Volocity Acquisition Suite (v6.3, Perkin Elmer). The percentage of cells with TDP-25 aggregates was quantified relative to total EGFP-positive cells across three fields of view (*n* ≥ 3, > 100 cells per replicate).

### Tissue lysis

Mice were anesthetized and transcardially perfused with ice-cold PBS, as above. Mouse brains were dissected and snap-frozen in liquid nitrogen. Whole hemi-brains were lysed in ice-cold low salt buffer (50 mM Tris, pH 7.5, 150 mM NaCl, 5mM EDTA) with a pre-cooled Dounce tissue grinder (Kimble). The tissue lysate was divided into two portions. 20% SDS was added to one portion the lysate to yield a final concentration of 2% SDS, the lysate was then sonicated and stored as total lysate for Western blots. Fractionation was performed on the second portion of tissue lysate. The lysate was centrifuged at 20,000 xg for 20 min at 4 °C. The supernatant was stored as the low salt fraction. High salt buffer (50 mM Tris, pH 7.5, 750 mM NaCl, 5 mM EDTA) was added to the pellet, mixed, and centrifuged again at 20,000 x g for 20 min at 4 °C. The supernatant was stored as the high salt fraction. High salt buffer with 1% Triton X-100 was added to the pellet, mixed, incubated for 5 min at room temperature, and centrifuged at 20,000 x g for 20 min at 4 °C. The supernatant was stored as the high salt with TX-100 fraction. High salt buffer with 30% sucrose was added to the pellet, mixed, and centrifuged at 20,000 xg for 20 min at 4 °C. This resulted in a myelin float; the supernatant was discarded, and the pellet was dissolved and sonicated in high salt buffer with 2% SDS. This final fraction was stored as the insoluble fraction. Halt protease and phosphatase inhibitors (Thermo fisher) were added to all buffers. The Pierce BCA Protein Assay (Thermo Fisher) was performed on all lysates to ensure comparable concentrations of lysates. Equal volumes for the fractions are then loaded onto 10% SDS-PAGE gels.

### Western blot analysis

3X SDS sample buffer (188 mM Tris-HCl, pH 6.8, 3% SDS, 30% glycerol, 0.01% bromophenol blue, 15% β-mercaptoethanol) was added to the lysates, and the samples were boiled for 5 min. The samples were separated on 10% SDS-PAGE gels (18% for LC3 blots) and transferred to polyvinylidene fluoride (PVDF) membranes (0.45 μm). The membranes were blocked with 5% skim milk in Tris-buffered saline with 0.1% Tween-20 (TBST) for 1 h at room temperature. For blots detecting phosphorylated proteins, membranes were blocked with 5% bovine serum albumin (BSA) in TBST. The membranes were then incubated with primary antibodies [anti-GAPDH (Abcam, 1:10,000); anti-p62 (Abcam, 1:2000); anti-LC3A/B (Abcam, 1:1000); anti-ULK1 (Cell Signaling, 1:1000)] overnight at 4 °C. Afterward, the membranes were washed with TBST and incubated with horseradish peroxidase (HRP)-conjugated secondary antibodies (Jackson ImmunoResearch, 1:5000) at room temperature for 2 h. The membranes were rewashed with TBST and treated with Western Lightning Plus Chemiluminescent Substrate (Perkin Elmer) before being developed onto film. The blots were then scanned and quantified by densitometric analysis using ImageJ.

### Statistical analysis

Statistical analysis was performed using Prism (v8.4.3, GraphPad), with the specific tests used listed in the figure legends. Differences were considered statistically significant when *p <.05, **p <.01, ***p <.001, ****p <.0001.

## Results

### AAV9-mediated expression of TDP-35 and TDP-25 in vivo

**Fig. 1:**
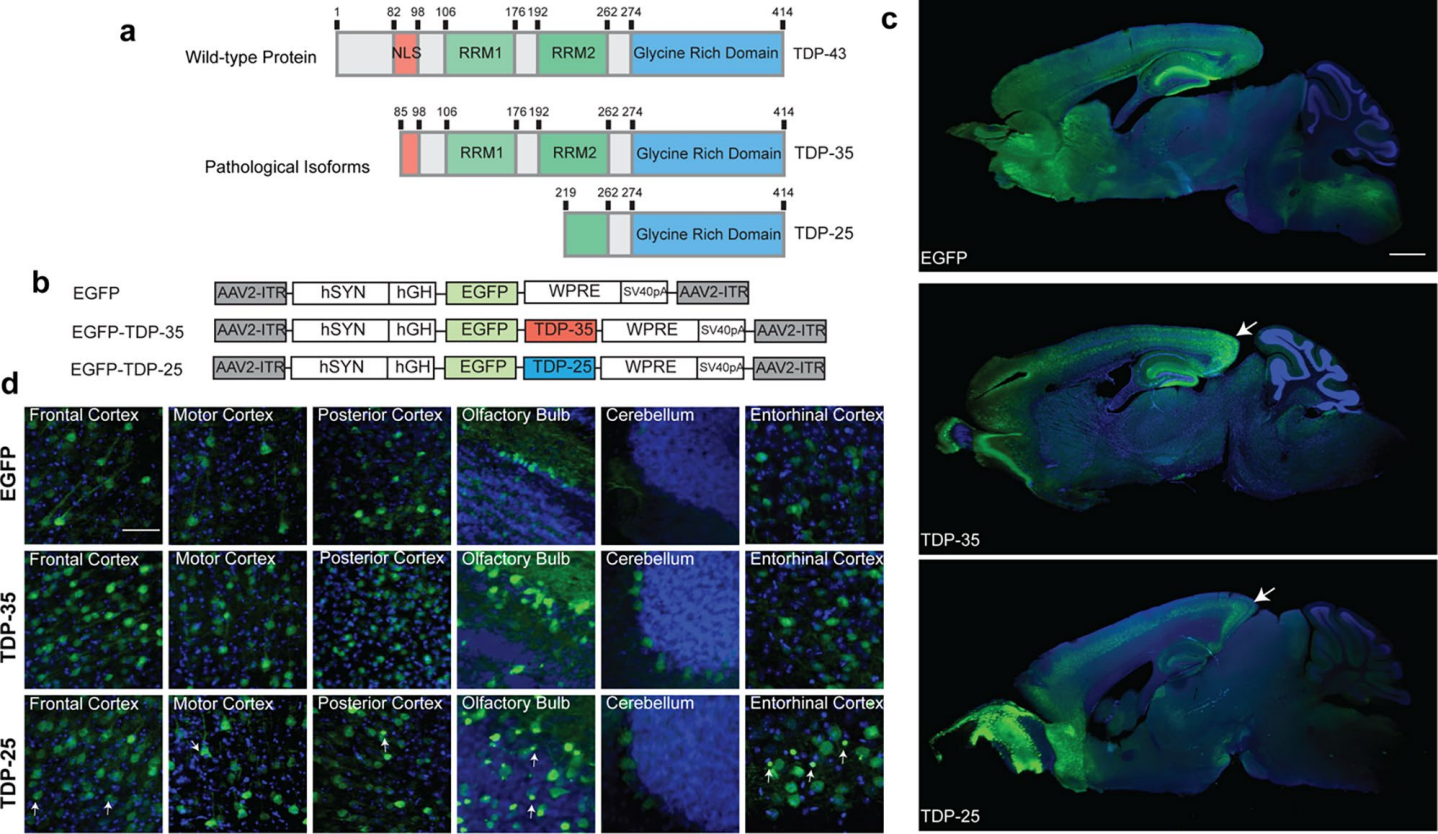
rAAV9-mediated expression of TDP-35 and TDP-25. **a** C-terminally truncated pathological isoforms of TDP-43, TDP-35 (aa 85–414) and TDP-25 (aa 219–414). **b** Recombinant adeno-associated viruses serotype 9 (rAAV9) vectors were generated to express EGFP (control), EGFP-TDP-35, or EGFP-TDP-25 under the human synapsin (hSYN1) promotor for neuron-specific expression. **c** Sagittal brain sections from 18-month-old mice labeled with EGFP antibody (green) and DAPI nuclear stain (blue). TDP-35 was widely expressed throughout the cortex, with highest expression in the hippocampus and posterior cortex (arrow). TDP-25 showed enriched expression in the olfactory bulb, layer V cortical neurons and the posterior cortex (arrow). **d** Higher magnification images showing EGFP, TDP-35 and TDP-25 expression in different brain regions (green). TDP-35 appeared diffusely distributed in the nucleus and cytoplasm. TDP-25 appeared diffusely and as distinct cytoplasmic aggregates, particularly in the olfactory bulb and entorhinal cortex and, to a lesser extent, in the frontal and motor cortices (arrows). Scale bars: c, 1 mm; **d**, 50 μm

 Ectopic expression of TDP-43 C-terminal fragments, TDP-35 (aa 85–414) and TDP-25 (aa 219–414; representing caspase-3 cleaved TDP-43 [26] (Fig. 1a), leads to the formation of cytoplasmic stress granules or phosphorylated, ubiquitinated, and p62 labelled cytoplasmic aggregates in cultured cells, respectively [27]. To create in vivo models, we generated recombinant adeno-associated viruses serotype 9 (rAAV9) vectors expressing EGFP-tagged TDP-35, EGFP-tagged TDP-25, and EGFP as a control (Fig. 1b). These viral vectors were designed to drive neuron-specific expression in vivo through the use of the human synapsin (hSYN1) promoter [25]. Intracerebroventricular (i.c.v.) injections of rAAV9 viral vectors expressing EGFP, EGFP-TDP-35, and EGFP-TDP-25 were administered to neonatal wild-type (WT) C57BL/6 mice at postnatal day 0 (P0). The mice were then aged to 6 and 18 months, forming three experimental groups: EGFP, EGFP-TDP-35 (TDP-35), and EGFP-TDP-25 (TDP-25) mice (Fig. 1c). Although hSYN1 drives neuronal expression of all three AAV9 constructs, variations in the regional expression of EGFP, TDP-35 and TDP-25 were apparent, with EGFP and TDP-35 exhibiting wide-spread expression throughout the cerebral cortex and hippocampus, and TDP-25 showing concentrated expression in layer V neurons of the cerebral cortex, low level of expression in the hippocampus, and high expression in the olfactory bulb (Fig. 1c). Higher magnification analysis across different brain regions revealed expression of EGFP, TDP-35 and TDP-25 in neurons of the frontal cortex, motor cortex, posterior cortex, olfactory bulb, and entorhinal cortex, with TDP-35 and TDP-25 but not EGFP expressed in Purkinje cells (Fig. 1d). TDP-35 appeared diffusely expressed in neurons in all brain regions examined. In contrast, TDP-25 formed abundant cytoplasmic aggregates in areas of high expression, including the posterior cortex, entorhinal cortex, and olfactory bulb (Fig. 1d, arrows), with occasional TDP-25 aggregates observed in other regions, including the frontal and motor cortices (Fig. 1d, arrows).

### TDP-35 and TDP-25 mice exhibit distinct behavioral phenotypes

**Fig. 2:**
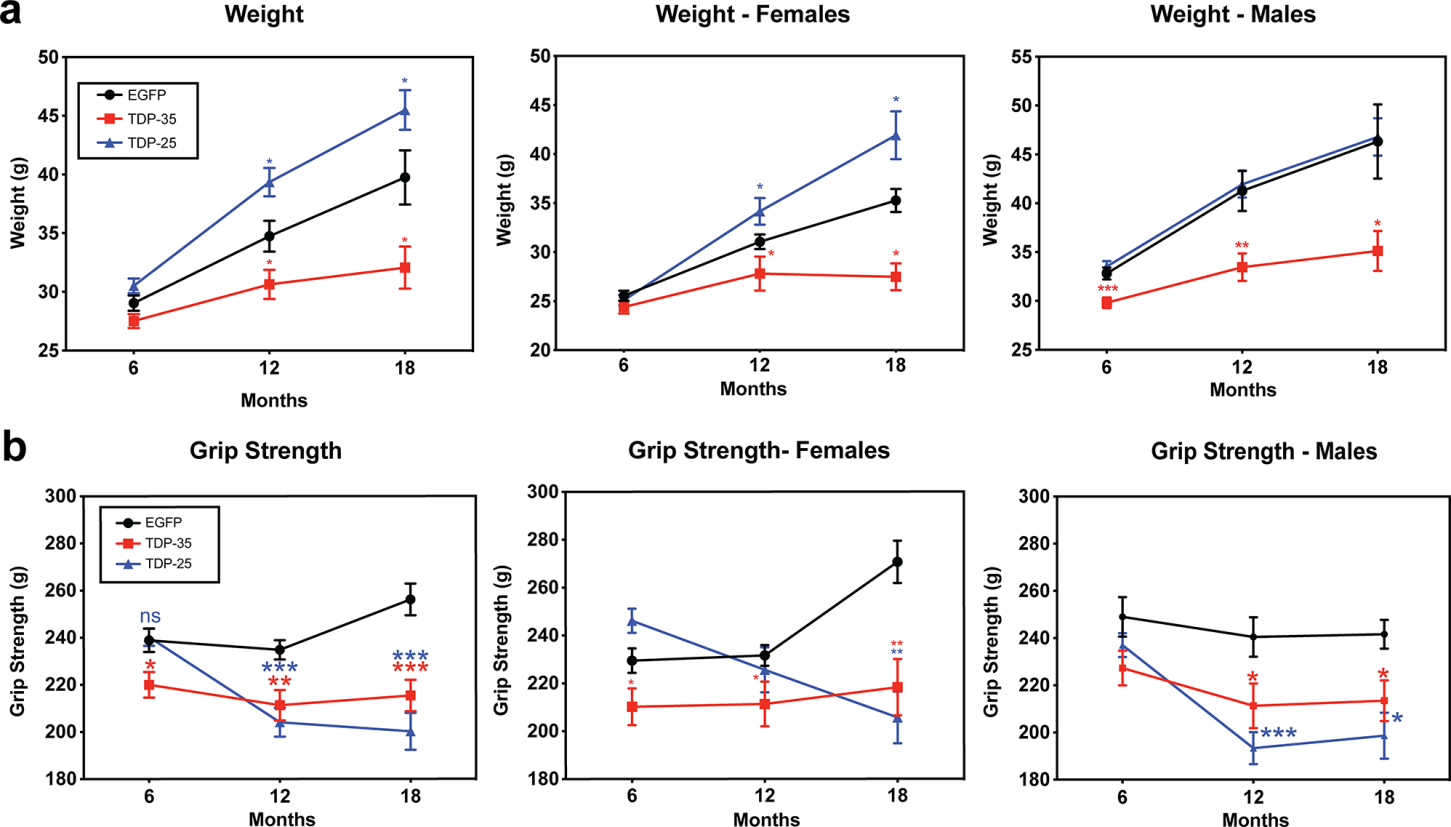
Weight differences and motor deficits in TDP-35 and TDP-25 mice. **a** TDP-35 expressing mice exhibited significantly lower body weight and stunted growth from 6 to 18 months of age compared to the EGFP control mice; TDP-25 mice were overall heavier than the control mice. Female and male mice followed the same trend. **b** TDP-35 mice show lower grip strength throughout 6 to 18 months; TDP-25 mice show a sharp decline in grip strength by 12 months of age. Data are presented as mean ± SEM. Statistical significance was determined using multiple t-tests with Holm-Sidak correction. Asterisks denote statistical significance: *P <.05, **P <.01, ***P <.001, ****P <.0001. Sample sizes: 6 months, n = 26 F/24 M (EGFP), 17 F/23 M (TDP-35), 25 F/45 M (TDP-25); 12 months, n = 16 F/9 M (EGFP), 12 F/12 M (TDP-35), 10 F/20 M (TDP-25); 18 months, n = 6 F/6 M (EGFP), 4 F/6 M (TDP-35), 4 F/14 M (TDP-25)

TDP-35 mice exhibited stunted growth from 6 to 18 months with lower body weight compared to EGFP control mice, whereas TDP-25 mice were, on average, heavier than EGFP mice, though this difference was less pronounced in male mice (Fig. 2a). Both TDP-25 and TDP-35 mice showed lower grip strength than EGFP mice by 12 months of age (Fig. 2b). However, while TDP-35 mice showed lower grip strength throughout the 6 to 18 months test period, TDP-25 mice exhibited a sharp decline in grip strength by 12 months of age, with further decline over the 18-month test period (Fig. 2b). This indicates that motor decline in TDP-25 mice was age-dependent, whereas TDP-35 mice had innate motor weaknesses.

**Fig. 3:**
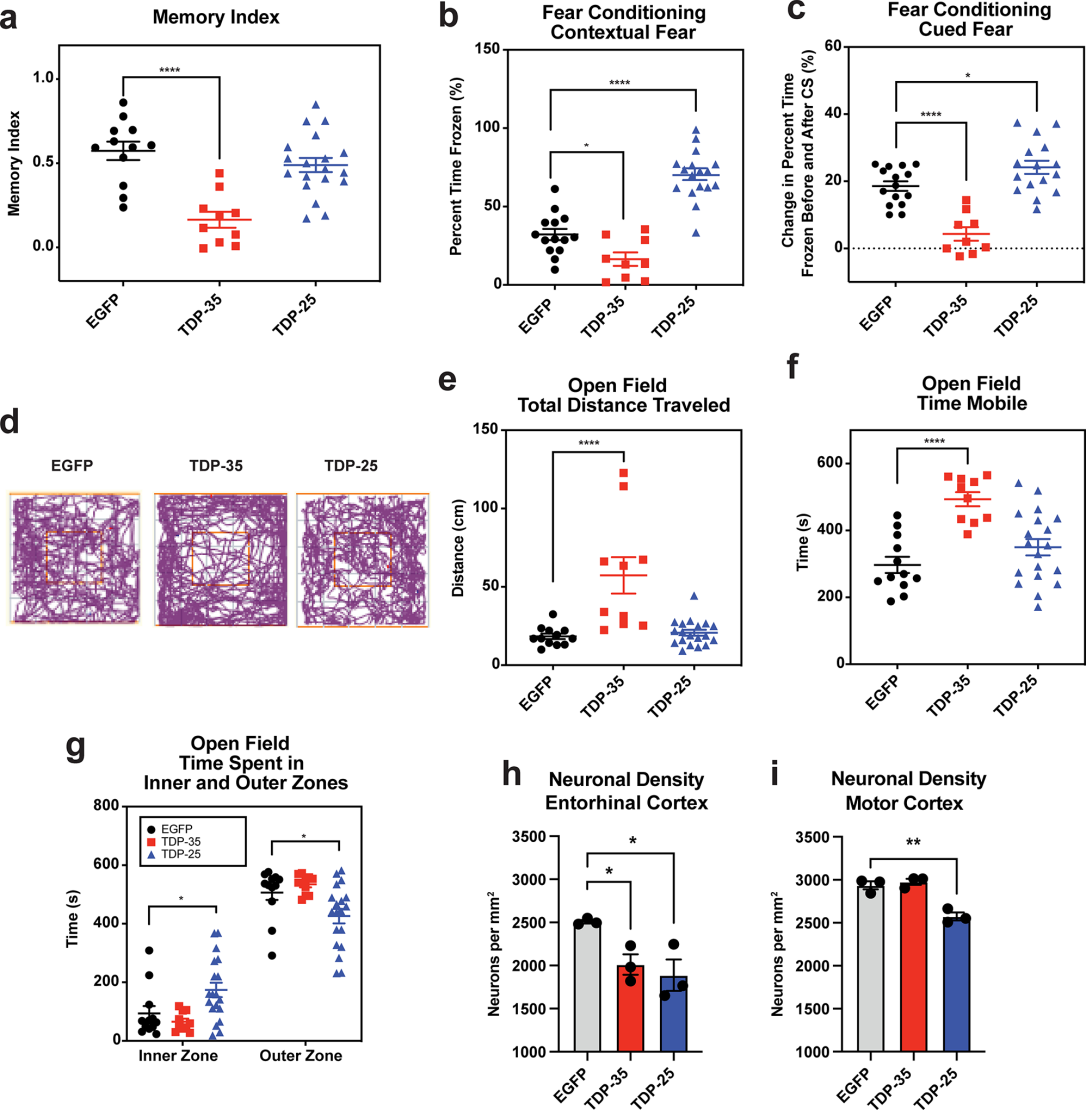
Cognitive differences and neuronal loss in TDP-35 and TDP-25 mice. **a** TDP-35 mice exhibited impaired recognition memory, reflected by a significantly lower memory index in the novel recognition (NOR) test. No memory impairment was observed in TDP-25 mice. TDP-35 mice demonstrated reduced freezing in both **b** contextual and **c** cued fear conditioning tests, consistent with impaired associative learning. In contrast, TDP-25 showed increased freezing in both paradigms. **d** Open-field testing revealed hyperactivity in TDP-35 mice, including increased **e** total distance traveled and **f** mobility time. No significant changes were observed in TDP-25 mice for distance or mobility; however, **g** TDP-25 mice spent more time in the center zones of the apparatus, suggesting altered anxiety-like behavior. Quantification of NeuN labelled brain sections revealed a reduction in neuronal density in the **h** entorhinal cortex of TDP-35 and TDP-25 mice and the **i** motor cortex of TDP-25 mice, relative to EGFP controls. Data are presented as mean ± SEM. Behavioral analyses were conducted in 18-month-old mice: EGFP (n = 6 F/6 M), TDP-35 (n = 4 F/6 M), TDP-25 (n = 4 F/14 M). Neuronal quantification was performed on n = 3 mice per group. Statistical significance was assessed using Kruskal-Wallis test (behavioral tests) and student’s t test (neuronal density). Asterisks denote significance: *P <.05, **P <.01, ***P <.001, ****P <.0001

Cognitive testing at 18 months of age demonstrated that TDP-35 mice had a lower memory index (MI) in novel object recognition (NOR) tasks (Fig. 3a, Supplementary Fig. 1a), less freezing in both contextual and cued fear conditioning (FC) tasks (Fig. 3b, c, Supplementary Fig. 1b-d), and hyperactivity and anxiety in open field (OF) tasks compared to EGFP mice (Fig. 3d-g, Supplementary Fig. 1e-g). In contrast, TDP-25 mice did not show impairment in NOR tasks (Fig. 3a) but exhibited more freezing in FC tasks (Fig. 3b, c) and spent significantly more time in the inner zones of the OF apparatus (Fig. 3g). This suggests that although TDP-25 mice showed enhanced fear responses in FC tasks, this did not appear to be due to increased anxiety. Next, we assessed if the expression of TDP-35 and TDP-25 induced neuronal loss, focusing on the motor cortex and entorhinal cortex, where high levels of expression were observed. Neuronal density was quantified from NeuN labelled brain sections from 18-month-old mice. Significant neuronal loss was observed in the entorhinal cortex of both TDP-35 and TDP-25 mice compared to EGFP mice (Fig. 3h), however, significant neuronal loss was observed in the motor cortex of TDP-25 mice but not in TDP-35 mice (Fig. 3i). Thus, the motor and cognitive phenotypes observed in TDP-25 and TDP-35 mice could be attributed, at least in part, to neuronal loss in the motor and entorhinal cortex.

### TDP-25 forms abnormally phosphorylated and ubiquitinated neuronal cytoplasmic aggregates in vivo, modelling TDP-43 pathology

**Fig. 4:**
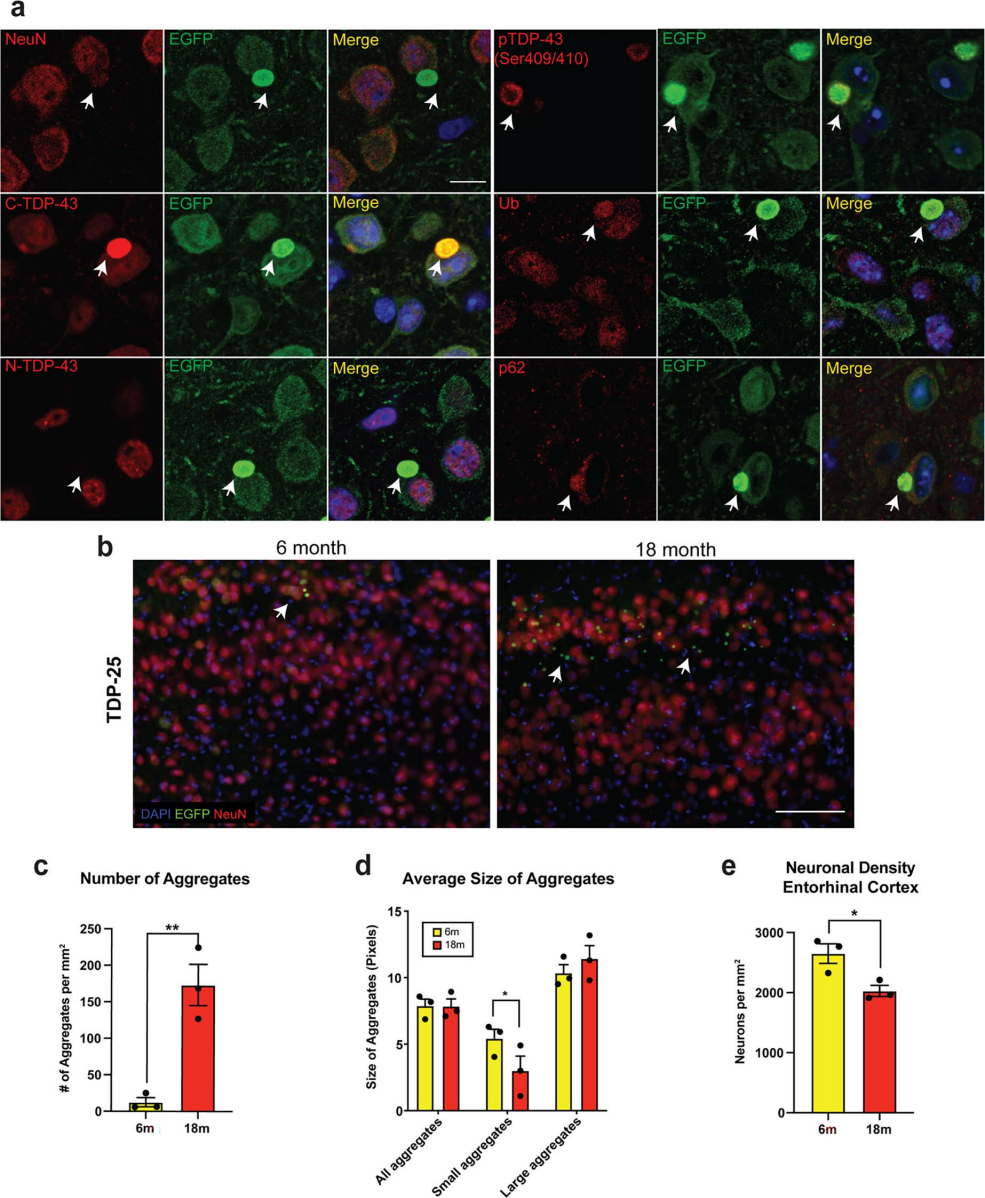
TDP-25 aggregates are abnormally phosphorylated, ubiquitinated and p62-positive. **a** Immunofluorescence images of the entorhinal cortex from TDP-25 mice show that cytoplasmic TDP-25 aggregates (EGFP-green) are localized to neurons (NeuN, red), and stain positive for C-terminal TDP-43 (C-TDP-43); and are negative for N-terminal TDP-43. TDP-25 aggregates stained positive for phosphorylated TDP-43 (Ser409/410), ubiquitin and p62. **b** Representative images of the entorhinal cortex of TDP-25 mice at 6 months and 18 months co-labelled with antibodies to EGFP (green) and NeuN (red). An increased number of TDP-25 aggregates is observed at 18 months compared to 6 months (arrows). **c** Quantification confirmed a significant increase in TDP-25 aggregates at 18 months relative to 6 months. **d** Aggregate size was also significantly larger in 18-month-old mice. **e** NeuN-based quantification of neuronal density revealed a significant reduction in neuron number at 18 months compared to 6 months, showing age-dependent neurodegeneration. Data are presented as mean ± SEM from n = 3 mice per group. Statistical significance was assessed using the Student’s t-test. Asterisks indicate significance levels: *P <.05, **P <.01, ***P <.001, ****P <.0001. Scale bars: a, 10 μm; b, 100 μm

Expression of TDP-25, but not TDP-35, generated cytoplasmic aggregates that appeared most abundant in the entorhinal cortex of TDP-25 mice (Fig. 1d). TDP-43 pathology and neuronal loss in the entorhinal cortex are associated with cognitive-behavioural impairments and psychiatric symptoms in the ALS/ FTD spectrum [28]. Neuronal expression of EGFP-TDP-25 was confirmed through NeuN staining, and the EGFP-TDP-25 aggregates stained positive for antibody to the C-terminal region of full-length TDP-43 (C-TDP-43), confirming that these aggregates contained TDP-25 (Fig. 4a). TDP-25 aggregates did not stain for endogenous mouse TDP-43 as shown using an antibody labelling the N-terminal region of TDP-43 (N-TDP-43), an epitope absent in TDP-25, indicating that cytoplasmic TDP-25 aggregates did not recruit endogenous mouse TDP-43 (Fig. 4a). Cytoplasmic TDP-25 aggregates were co-labelled with antibody to TDP-43 phosphorylated at serine 409/410 (pTDP-43), appearing as an outer shell, and were also labelled with ubiquitin and p62 (Fig. 4a), recapitulating hallmarks of TDP-43 pathology in ALS/FTD. As described above, TDP-25 mice exhibited a marked decline in grip strength by 12 months of age (Fig. 2b), while no significant difference was observed at 6 months compared to EGFP controls, indicating an age-dependent progression of neurodegeneration. We therefore sought to compare the number of TDP-25 aggregates and the extent of neuronal loss in 6-month-old and 18-month-old TDP-25 mice, specifically in the entorhinal cortex where TDP-25 aggregation was predominant. There was a ~ 150-fold increase in the number of TDP-25 aggregates in 18-month-old TDP-25 mice compared to 6-month-old mice (Fig. 4c), with a reduction in the number of smaller aggregates (defined by the 10th percentile size-wise of all aggregates) (Fig. 4d). Quantification of neuronal density using NeuN labelling revealed a significant reduction in neurons in 18-month-old TDP-25 mice compared to 6-month-old mice (Fig. 4e).

### Loss of C9orf72 causes autophagic deficits and exacerbates TDP-25 aggregation

**Fig. 5:**
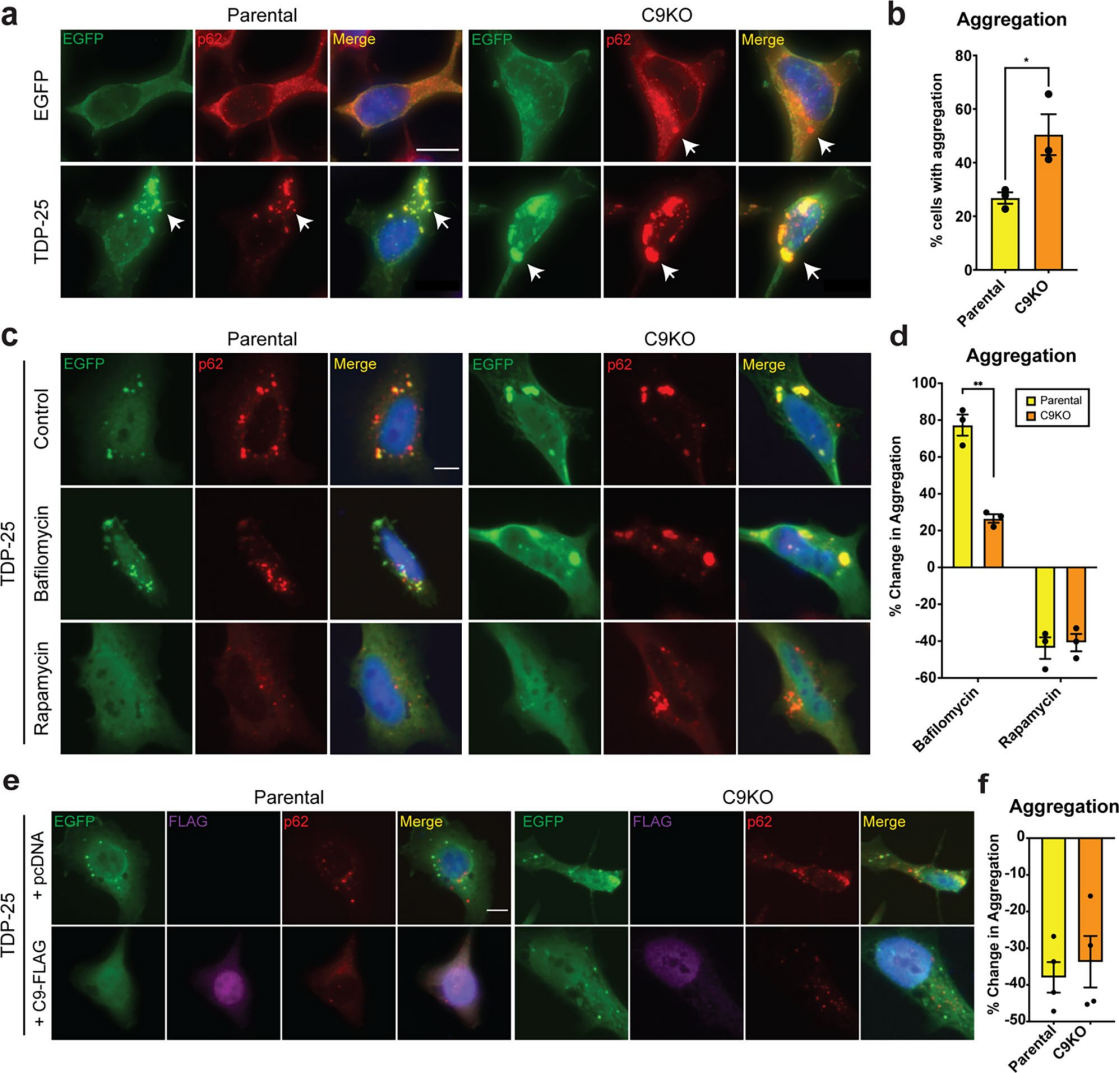
TDP-25 aggregation is exacerbated by loss of C9orf72 in vitro. **a** Parental and C9KO HeLa cells ectopically expressing EGFP-TDP-25 show cytoplasmic TDP-25 aggregates (green) co-labeled with p62 (red); DAPI nuclear stain (blue). TDP-25 aggregates are larger in the C9KO cells than parental controls (arrows). **b** Quantification confirmed that TDP-25 aggregates are significantly increased in C9KO cells compared to controls. **c** Inhibition of autophagy with bafilomycin led to increased TDP-25 aggregates in parental cells and a more modest increase in C9KO cells. Activation of autophagy with rapamycin reduced the number of TDP-25 aggregates in both parental and C9KO cells. **d** Quantification revealed that bafilomycin treatment increased TDP-25 aggregates by 77% in parental cells and 26% in C9KO cells. Rapamycin treatment reduced TDP-25 aggregates by 40% in both parental and C9KO cells. **e** Ectopic expression of FLAG-tagged C9orf72 (purple) significantly reduced TDP-25 aggregation in both parental and C9KO cells. **f** Quantification shows that exogenous expression of C9orf72 reduced TDP-25 aggregates by ~ 35% in both parental and C9KO cells. Data are presented as mean ± SEM from ≥ 3 independent experiments. Statistical significance was determined using Student’s t test. Asterisks denote statistical significance: *P <.05, **P <.01, ***P <.001, ****P <.0001. Scale bar: 10 μm

TDP-43 pathology is characteristic of C9-ALS/FTD cases [29]. We first investigated if loss of C9orf72 would exacerbate TDP-25 aggregation in cell culture. EGFP-TDP-25 was expressed in *C9orf72*-knock out (C9KO) HeLa cells and parental control cells using transient transfection. TDP-25 formed visibly larger aggregates in C9KO cells compared to the parental cell line (Fig. 5a), with quantification revealing a 26% increase in the number of cells with TDP-25 aggregates in C9KO cells versus the parental control cells (Fig. 5b). In line with previous studies and our findings in TDP-25 mice (Fig. 4a), TDP-25 aggregates stained positive for p62 (Fig. 5a) [26, 27]. Also known as Sequestome 1 (SQSTM1), p62 is a selective autophagy receptor, linking ubiquitinated cargo to the autophagosome membrane for lysosomal degradation [30, 31]. The accumulation of cytoplasmic p62 puncta in neurons is a hallmark of impaired autophagy [32]. C9orf72 is reported to have roles in the initiation of autophagy, and its loss is associated with autophagic deficits (reviewed in [33]). Supporting this, increased levels of cytoplasmic p62 puncta were observed in C9KO cells compared to the parental control cells (Fig. 4a, Supplementary Fig. 2a, b). We examined whether autophagy modifiers bafilomycin (autophagy inhibitor) and rapamycin (autophagy activator) would exacerbate or rescue TDP-25 aggregation in parental and C9KO cells (Fig. 5c, d, Supplementary Fig. 2). Bafilomycin treatment increased the abundance of cytoplasmic TDP-25 aggregates by 77% in parental cells, and by 26% in C9KO cells (Fig. 5c, d). This suggests that existing autophagic impairment in C9KO cells limits the effect of further autophagy inhibition. In contrast, rapamycin reduced TDP-25 aggregation by 40–50% in both the parental and C9KO cells (Fig. 5c, d). We also examined whether co-expression of C9orf72 with TDP-25 could rescue TDP-25 aggregation (Fig. 5e, f). Co-expression led to a 40–50% reduction in both parental and C9KO cells and was accompanied by more diffuse cytoplasmic p62 staining, indicating that exogenous C9orf72 expression mitigates TDP-25 aggregation and improves autophagic function (Fig. 5e, f).

### C9orf72-deficient mice expressing TDP-25 show motor deficits and neuronal loss in the entorhinal cortex and motor cortex

**Fig. 6:**
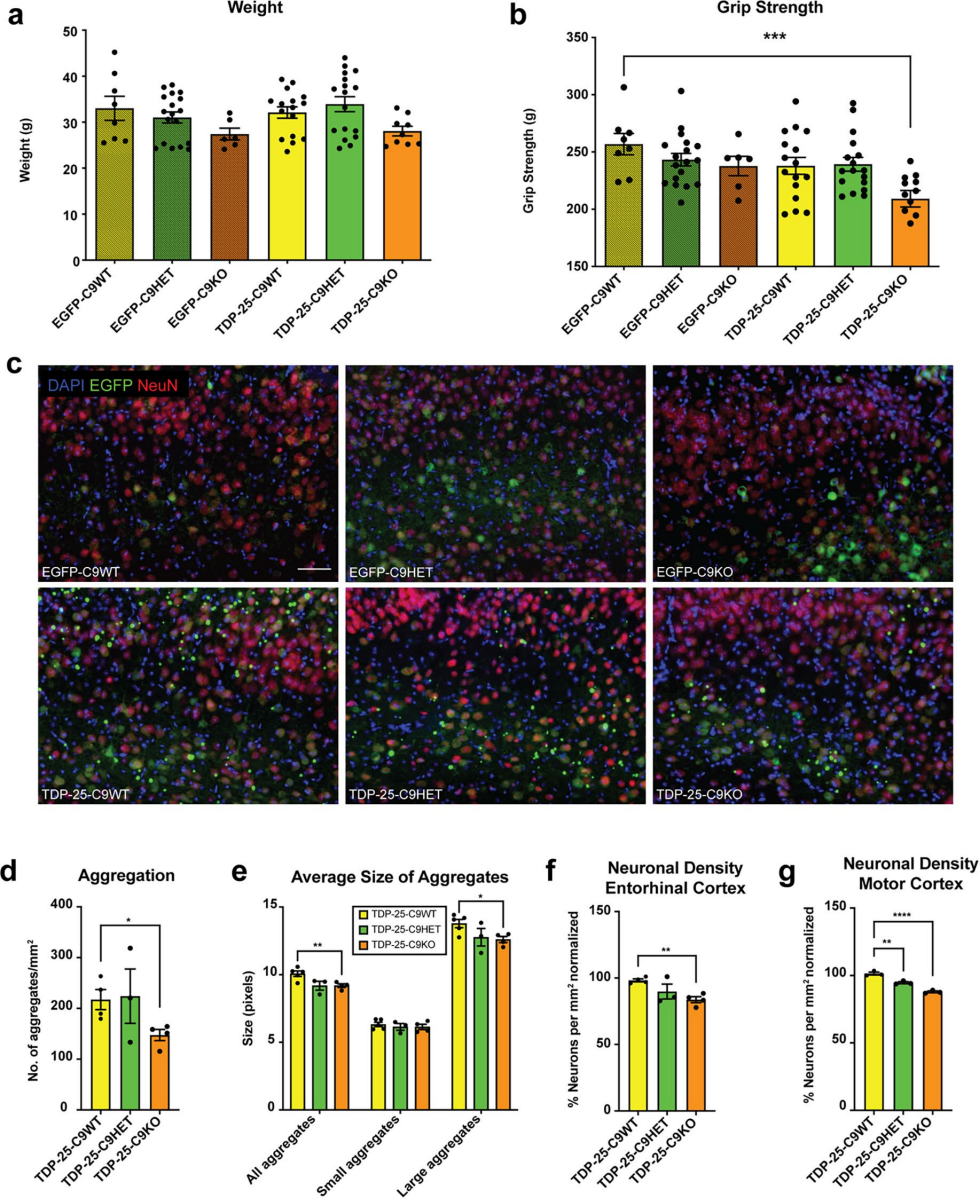
Loss of C9orf72 induces motor deficits and neuronal loss in the entorhinal cortex and motor cortex of 8-month-old TDP-25 mice.** a** Body weight measurements at 8 months of age showed no significant differences across genotypes. **b** Grip strength testing revealed significantly reduced grip strength in TDP-25-C9-KO mice compared to controls, indicating motor impairment. Sample sizes: EGFP-C9WT (*n* = 8), EGFP-HET (*n* = 18), EGFP-C9KO (*n* = 6), TDP-25-C9WT (*n* = 16), TDP-25-C9HET (*n* = 17), TDP-25-C9KO (*n* = 13). **c** Representative images of the entorhinal cortex labeled EGFP (green), NeuN (red), DAPI (blue) show fewer TDP-25 aggregates in TDP-25-C9KO mice compared to TDP-25-C9WT mice. **d** Quantification confirmed a significant reduction in TDP-25 aggregates in TDP-25-C9KO mice. **e** The median size of TDP-25 aggregates, as well as the average size of larger aggregates, was significantly smaller in TDP-25-C9KO mice compared to TDP-25-C9WT mice, with TDP-25 aggregates in TDP-25-C9HET trending in the same direction. **f** Neuronal density was significantly reduced in TDP-25-C9KO mice compared to TDP-25-C9WT in the entorhinal cortex and **g** significant neuronal loss was observed in the motor cortex of both TDP-25-C9HET and TDP-25-C9KO mice compared to TDP-25-C9WT mice. Data are presented as mean ± SEM from ≥ 3 independent animals per group. Statistical significance was determined using Kruskal-Wallis test (behavioral tests) and Mann-Whitney test (quantification). Asterisks denote significance: *P <.05, **P <.01, ***P <.001, ****P <.0001. Scale bar in **c**: 100 μm

As loss of C9orf72 exacerbated TDP-25 aggregation in vitro, we next investigated whether C9orf72 deficiency would affect TDP-25 aggregation in vivo. Heterozygous *C9orf72* KO (C9HET), homozygous *C9orf72* KO (C9KO) and wildtype control littermate (C9WT) P0 pups were i.c.v. injected with AAV9-EGFP-TDP-25 or AAV9- EGFP, as described above, and aged to 8 months. Mice were analyzed at 8 months to preclude confounding factors of peripheral immune phenotypes that become apparent in C9KO mice by 10–12 months of age [23, 34–37]. Biochemical fractionation of total mouse brain lysates using buffers of increasing stringencies showed successful transgene expression and that TDP-25 partitioned mainly to the insoluble fraction in WT and C9orf72-deficient mice (Supplementary Fig. 3). No significant weight differences were observed between the different cohorts (Fig. 6a, Supplementary Fig. 4a). In contrast to TDP-25-WT mice, which exhibited reduced grip strength by 12 months of age (Fig. 2b), TDP-25-C9KO mice show significantly decreased grip strength at 8 months, indicating that C9orf72 loss accelerates motor impairments in TDP-25 expressing mice (Fig. 6b, Supplementary Fig. 4b). TDP-25 exhibited similar brain region expression patterns in TDP-25-C9WT, TDP-25-C9HET and TDP-25-C9KO mice, with highest expression in layer V neurons of the cerebral cortex and the entorhinal cortex (Supplementary Fig. 4c). Neuronal cytoplasmic TDP-25 aggregates were highly abundant in the entorhinal cortex of all genotypes, and rarely observed in other brain regions, including the motor cortex, where TDP-25 expression levels were relatively lower (Fig. 6c). Quantification revealed a significant reduction in the number of TDP-25 aggregates in the entorhinal cortex of TDP-25-C9KO mice compared to TDP-25-C9HET and TDP-25-C9WT mice (Fig. 6d), and that the average size of aggregates was reduced in TDP-25-C9HET and TDP-25-C9KO mice compared to TDP-25-C9WT mice (Fig. 6e). Assessment of neuronal density in the entorhinal cortex revealed ~ 2% neuronal loss in TDP-25-WT mice, ~ 10% in TDP-25-C9HET mice and ~ 16% in TDP-25-C9KO mice, when compared to their respective controls (EGFP-WT, EGFP-C9HET, EGFP-C9KO) (Fig. 6f; Supplementary Fig. 4d). Similar results were observed in the motor cortex, with 5% neuronal loss in TDP-25-C9HET mice and 11% in TDP-25-C9KO mice, both significantly higher neuronal loss than observed in TDP-25-C9WT mice (Fig. 6g, Supplementary Fig. 4e). These results indicate a gene dosage effect of *C9orf72* on neuronal loss in TDP-25 expressing mice. Notably, this neuronal loss occurred in the absence of overt neuroinflammation, as no significant increases in microgliosis or astrogliosis were detected by Iba-1 or GFAP staining, respectively (Supplementary Fig. 5). Collectively, these findings demonstrate that loss of C9orf72 exacerbates motor deficits and neuronal loss in TDP-25 expressing mice. Although the TDP-25 aggregate burden was reduced in TDP-25-C9KO mice, this was accompanied by increased neuronal loss. A Spearman correlation analysis between TDP-25 aggregate number and neuronal density in the TDP-25 mice of all genotypes revealed a moderate positive correlation (*r* =.6182, *p* =.0478) between neuronal density and aggregation, suggesting that neurons harbouring aggregates may have degenerated.

### Autophagic deficits in C9orf72 deficient mice expressing TDP-25

**Fig. 7:**
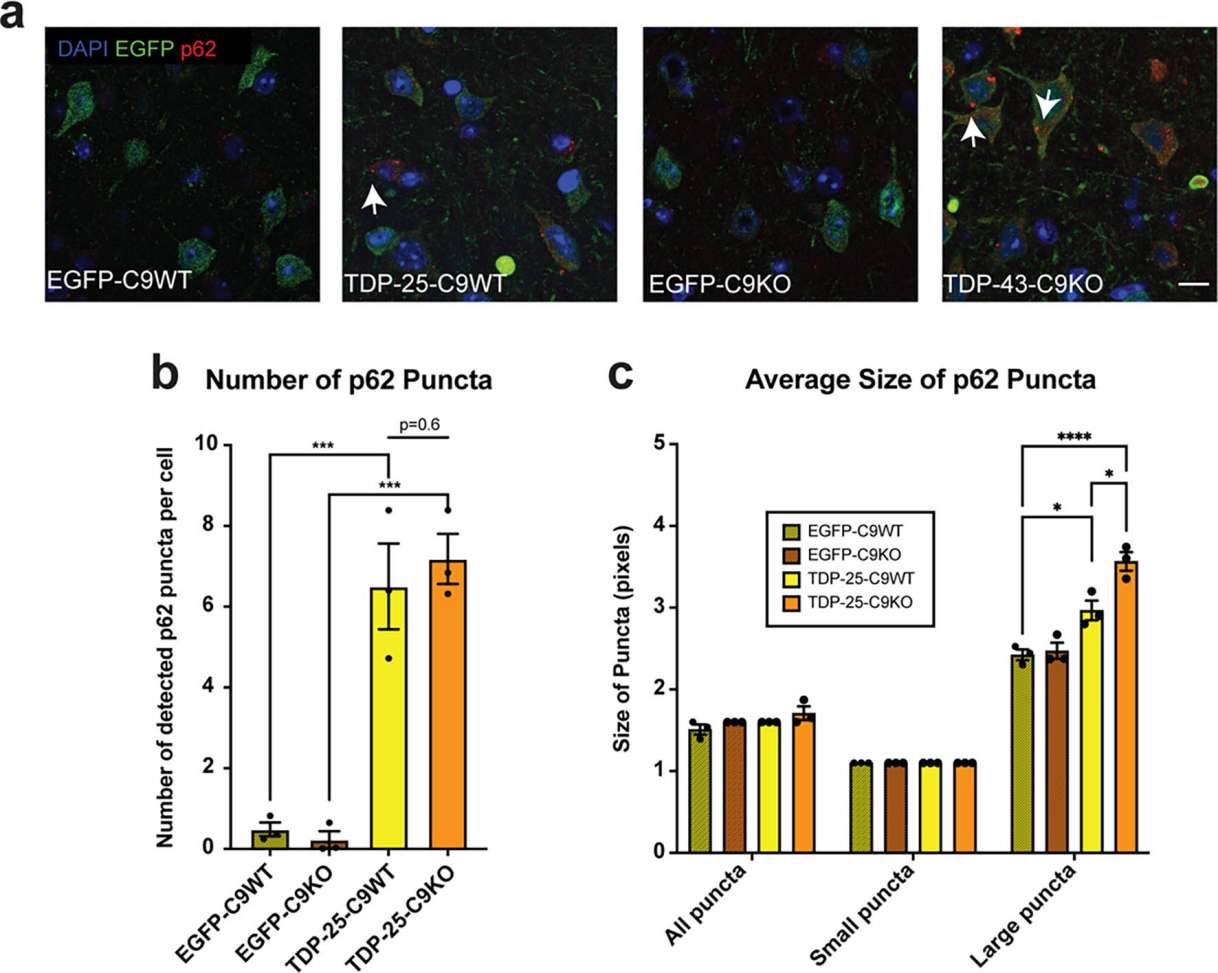
Englarged neuronal p62 puncta in TDP-25-C9KO mice. **a** Representative images of the entorhinal cortex showing increased p62 puncta (red) in EGFP-positive (green) neurons of TDP-25 expressing mice compared to EGFP controls (DAPI, blue). TDP-25 aggregates co-labeled with p62 (yellow). More abundant and larger p62 puncta were observed in TDP-25-C9KO neurons compared to TDP-25-C9WT neurons (arrows). DAPI (blue). **b** Quantification confirmed a significant increase in neuronal p62 puncta in TDP-25 expressing mice compared to EGFP controls, with a trend toward further elevation in TDP-25-C9KO neurons compared to TDP-25-C9WT neurons. **c** The average size of p62 puncta was greater in TDP-25-C9KO neurons compared to TDP-25-C9WT neurons. Data represent mean ± SEM from three biological replicates. Statistical significance was determined using the Student’s t test. Asterisks denote significance levels: *P <.05, **P <.01, ***P <.001, ****P <.0001. Scale bar in A: 10 μm

**Fig. 8:**
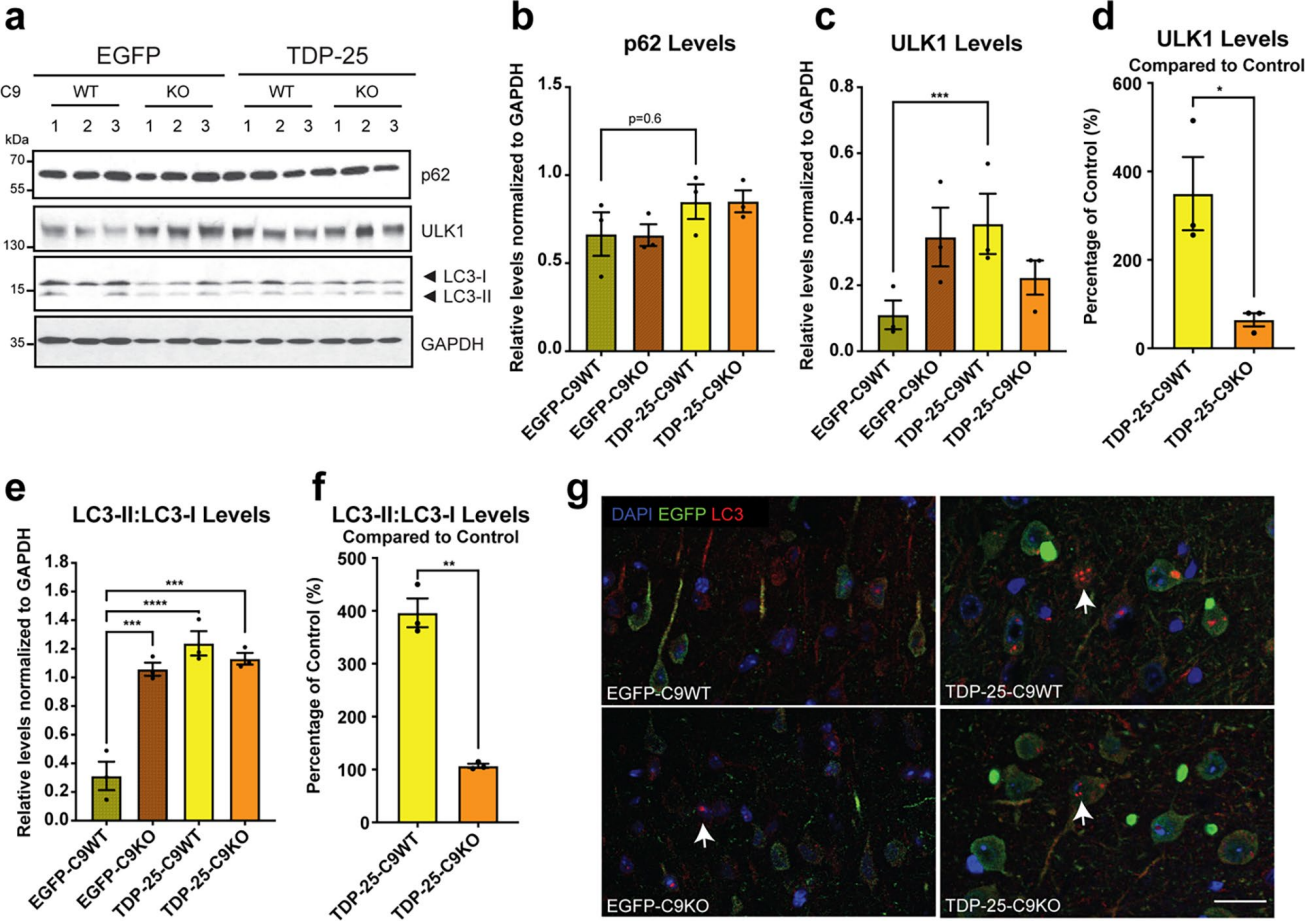
Changes in autophagy markers TDP-25 expressing mice. **a** Western blot analysis of autophagy-related proteins (p62, ULK1, LC3I and LC3II) in brain lysates in EGFP-WT, EGFP-C9KO, TDP-25-C9WT and TDP-25-C9KO mice, using GAPDH as loading control. **b** No significant changes in p62 protein levels were observed across genotypes. **c** ULK1 levels were significantly elevated in TDP-25-C9WT mice. **d** ULK1 levels were elevated 3.5-fold in TDP-25-C9WT mice and 0.75-fold in TDP-25-C9KO mice when normalized to their respective EGFP-WT and EGFP-C9KO controls. **e** LC3II: LC3I levels were significantly elevated in EGFP-C9KO, TDP-25-C9WT and TDP-25-C9KO mice compared to EGFP-WT. **f** LC3II: LC3I levels were elevated 4-fold in TDP-25-C9WT mice and only marginally increased in TDP-25-C9KO mice when normalized to their respective EGFP-WT and EGFP-C9KO controls. **g** Representative immunofluorescence images of the entorhinal cortex showing LC3 puncta (red) in EGFP-C9KO, TDP-25-C9WT and TDP-25-C9KO mice (DAPI, blue). LC3 puncta did not co-localize with TDP-25 aggregates (green). Data are presented as mean ± SEM from three biological replicates. Statistical significance was determined using the Student’s t test. Asterisks denote significance levels: *P <.05, **P <.01, ***P <.001, ****P <.0001. Scale bar in g: 10 μm

As described above, the accumulation of cytoplasmic p62 puncta is a hallmark of impaired autophagy and protein aggregation. Co-immunofluorescence staining of the entorhinal cortex for EGFP and p62 revealed few p62-positive neuronal cytoplasmic puncta in neurons of EGFP-C9WT and EGFP-C9KO mice, with no significant differences between genotypes (Fig. 7a). In contrast, TDP-25-C9WT mice exhibited a more significant number and size of neuronal p62 puncta, which were further increased in TDP-25-C9KO mice, suggesting that C9orf72 deficiency further impaired the autophagic response to TDP-25 expression (Fig. 7b, c). Biochemical analysis of total brain lysates showed a trend towards increased levels of p62 in both TDP-25-C9WT and TDP-25-C9KO mice compared to the respective EGFP-expressing control mice (Fig. 8a, b). C9orf72 has been shown to interact with ULK1 to initiate autophagy [14, 15]. To explore potential changes in autophagy initiation, we measured ULK1 levels in the different mouse cohorts. We observed a 3.5-fold increase in ULK1 levels in EGFP-C9KO mice compared to EGFP-C9WT mice (Fig. 8a, c). ULK1 levels were significantly increased in TDP-25-C9WT mice, with less elevation observed in TDP-25-C9KO mice (Fig. 8c). When normalized to their respective EGFP controls, TDP-25-C9WT mice exhibited a 3.5-fold higher ULK1 expression than TDP-25-C9KO mice (Fig. 8d). Thus, although the loss of C9orf72 and expression of TDP-25 independently upregulate ULK1 levels, there is reduced ULK1 elevation in the context of both C9orf72 loss and TDP-25 expression. This indicates that C9orf72 deficiency attenuates the autophagic response to TDP-25 expression. We also assessed LC3 (microtubule-associated protein 1 light chain 3) levels to evaluate autophagic flux. During autophagy, LC3-I conjugates with phosphatidylethanolamine to form LC3-II, promoting autophagosome expansion. After fusion with lysosomes, LC3-II is degraded or recycled to LC3-I. An increased LC3-II: LC3-I ratio reflects autophagosome accumulation or impaired autophagic flux [38]. LC3-II: LC3-I ratios were elevated in brain lysates from EGFP-C9KO, TDP-25-C9WT, and TDP-25-C9KO mice compared to EGFP-C9WT controls (Fig. 8e). When normalized to their respective EGFP controls, TDP-25-C9WT mice exhibited a 4-fold increase in LC3-II: LC3-I ratios, whereas TDP-25-C9KO mice showed only a modest increase (Fig. 8f). This finding was supported by immunofluorescence labelling, showing higher numbers of LC3 puncta in neurons of the entorhinal cortex in TDP-25-C9WT mice compared to TDP-25-C9KO mice (Fig. 8g). These results suggest that while TDP-25 expression induces autophagosome formation in TDP-25-C9WT mice, this autophagic response is impaired in TDP-25-C9KO mice. Collectively, our findings demonstrate that TDP-25 expression activates autophagy, as indicated by increased levels of p62, ULK1, and LC3-II, and that this response is impaired by loss of C9orf72.

## Discussion

Cytoplasmic aggregation of TDP-43 is a key pathology linking ALS and FTD. Emerging evidence suggests impaired protein clearance mechanisms, including autophagy, contribute to TDP-43 accumulation and associated neuronal loss in disease. *C9orf72*, repeat expansions in which are the most common genetic cause of ALS/FTD, plays a role in autophagy regulation. C9orf72 protein levels are reduced in C9-ALS/FTD, and in this study, we investigated the impact of C9orf72 deficiency on TDP-43 aggregation and neurodegeneration in TDP-25 models, both in vitro and in vivo. We demonstrate that loss of C9orf72 leads to impaired autophagy, increased TDP-25 aggregation in vitro, and exacerbates motor deficits and neuronal loss in TDP-25-expressing mice in vivo. These findings support that autophagic deficits caused by C9orf72 contribute to TDP-43 pathology and neurodegeneration in C9-ALS/FTD.

To establish in vivo models, we used ectopic expression of pathologically relevant C-terminal fragments, TDP-35 and TDP-25. TDP-35 and TDP-25 lack the complete nuclear localization sequence (NLS) present in full-length TDP-43 and exhibit cytoplasmic localization in transfected cells, forming stress granules or abnormally phosphorylated and ubiquitinated aggregates labelled with p62, respectively [27]. We used rAAV9 vectors to drive neuron-specific expression of EGFP-tagged TDP-35 and TDP-25 in neonatal mice using the hSYN1 promoter. Mice were then aged to characterize age-dependent effects using immunohistochemical, behavioral, and biochemical analyses. Despite being expressed from the same vectors, TDP-35 and TDP-25 exhibited differences in brain region expression patterns, with TDP-35 showing expression throughout the cortex and TDP-25 expressed predominantly in layer V neurons and the entorhinal cortex. Several factors may have contributed to the regional variation in TDP-35 and TDP-25 expression levels, including their differing solubility and dynamic properties, which could influence their degradation patterns within different neuronal populations. While TDP-35 has been shown to form cytoplasmic stress granules when expressed in vitro [27], these were not observed in vivo, with TDP-35 showing diffuse nuclear and cytoplasmic expression in neurons. In contrast, TDP-25 recapitulated the in vitro phenotypes with abundant cytoplasmic aggregates, particularly in the entorhinal cortex, which were abnormally phosphorylated and labelled with ubiquitin and p62, modelling TDP-43 pathology in disease. Recent studies have shown that cells treated with fibrils encompassing the low complexity domain (LCD) of TDP-43 seed cytoplasmic aggregates that recruit endogenous nuclear TDP-43 [39, 40]. Although both TDP-35 and TDP-25 contain the LCD, we did not observe recruitment of nuclear TDP-43 to cytoplasmic TDP-35 (not shown) or TDP-25 in vitro or in vivo. This discrepancy may be due to TDP-35 and TDP-25 lacking the structural or surface properties required to recruit full-length TDP-43 or the delivery method, with LCD-fibrils added exogenously [39, 40], whereas TDP-35 and TDP-25 in the current study were expressed ectopically. Thus, expression of TDP-35 and, in particular, TDP-25 model TDP-43 aggregation independent of nuclear egress of the full-length protein. TDP-35 and TDP-25 mice exhibited differences in behavioural phenotypes, with TDP-35 showing innate motor weakness and TDP-25 mice showing an age-dependent decline in motor function. Additionally, TDP-35 mice and TDP-25 mice differed in cognitive tests, with TDP-35 mice demonstrating deficits in recognition memory and associative fear learning and hyperactivity in open-field tests. In contrast, TDP-25 mice exhibited increased freezing in fear conditioning tests, indicative of enhanced associative learning and emotional reactivity. These behavioural differences may be attributed to the variance in brain region expression or specific properties of TDP-35 and TDP-25. Notably, neuronal loss was observed in the entorhinal cortex of both TDP-35 and TDP-25 mice. The entorhinal cortex plays a significant role in fear conditioning, bridging sensory inputs and the amygdala [41]. The entorhinal cortex is affected in ALS/FTD, showing atrophy, neuronal loss, and TDP-43 pathology, potentially contributing to cognitive and psychiatric symptoms [28]. As such, neuronal loss in the entorhinal cortex may underpin fear-related phenotypes observed in TDP-35 and TDP-25 mice. Notably, neuronal loss in TDP-35 mice occurred in the absence of visible aggregates or stress granules, indicating that non-aggregated forms of TDP-35 are neurotoxic in vivo.

Since TDP-25 gave robust cytoplasmic aggregates, we focused on this model to study the effects of C9orf72 deficiency and effects on autophagy in exacerbating aggregation and behavioural phenotypes in vivo. Similar to findings in C9orf72 hexanucleotide repeat expressing mice, loss of C9orf72 led to exacerbated phenotypes in TDP-25 C9-KO mice [19, 21], causing motor deficits at 8 months instead of 12 months, as observed in TDP-25-C9WT mice. Interestingly, there were reduced numbers of cytoplasmic TDP-25 aggregates in TDP-25-C9HET and TDP-25-C9KO mice compared to TDP-25-C9WT mice, which may be attributed to the increased neuronal loss observed in C9orf72-deficient mice. C9orf72 in complex with SMCR8 has been shown to regulate autophagy at multiple levels, including autophagy initiation, autophagosome maturation and lysosome fusion (reviewed in [33]). Effects, however, are highly variable and dependent on cell type and environmental contexts, with, for example, loss of C9orf72 shown both to promote and impair autophagy initiation in different experimental systems (reviewed in [33]). We confirmed that loss of C9orf72 caused autophagic deficits in C9KO HeLa cells, as evidenced by increased cytoplasmic p62 puncta, leading to increased TDP-25 aggregation. These effects were rescued with rapamycin and exogenous expression of C9orf72. Thus, this evidence supports that loss of C9orf72 exacerbates TDP-25 aggregation through impaired autophagy. In contrast to C9KO HeLa cells, p62 puncta were not increased in neurons of C9KO mice. This is consistent with a previous study in which no changes in basal autophagy were observed in cortical tissue of C9orf72 deficient mice [21]. In addition to co-labelling of TDP-25 aggregates with p62, there was an overall increase in cytoplasmic p62 puncta in neurons of TDP-25-C9WT mice, likely due to proteotoxic stress induced by TDP-25 expression, which was further elevated in TDP-25-C9KO mice. This suggests a combinatorial effect in which loss of C9orf72 exacerbated autophagic deficits in TDP-25 expressing mice.

A critical regulator of autophagy initiation is the ULK1 complex, comprising ULK1, FIP200, ATG13 and ATG101, and increased ULK1 expression levels indicate autophagy activation. ULK1 activity is negatively regulated by mTOR, and previous studies suggest that C9orf72 can modulate autophagy by interacting with both ULK1 [14, 15] and mTOR [16, 17], acting as an activator or suppressor of autophagy initiation, respectively. Previous studies have shown that loss of the C9orf72-SMCR8 complex can lead either to upregulation or downregulation of ULK1 levels [42, 43], highlighting the context-dependent and multifaceted role of C9orf72 in autophagy regulation. We found that ULK1 levels were elevated 3.5-fold in C9KO mice and 4-fold in TDP-25-C9WT mice, indicative of autophagy initiation. However, no significant change was observed between C9KO and TDP-25-KO mice. Similarly, although LC3II: LCI ratios were increased in C9KO mice and TDP-25-C9WT mice, indicative of increased autophagosome formation or reduced autophagic flux, there was no significant difference between C9KO mice and TDP-25-KO mice. These findings suggest that loss of C9orf72 impairs the autophagic response to TDP-25 expression, which may contribute to exacerbated motor deficits and neuronal loss in observed TDP-25-KO mice.

In conclusion, our findings suggest that loss of C9orf72 impairs the autophagic response to cytoplasmically mislocalized and aggregated TDP-43, contributing to neurodegeneration in C9-ALS/FTD.

## Electronic supplementary material

Below is the link to the electronic supplementary material.

Supplementary Material 1

Supplementary Material 2

## Data Availability

No datasets were generated or analysed during the current study.
