# Supplementary material for: C9orf72 deficiency impairs the autophagic response to aggregated TDP-25 and exacerbates TDP-25-mediated neurodegeneration in vivo
Source: Acta Neuropathol Commun. 2025 Jun 28;13:136. doi: 10.1186/s40478-025-02061-5 (PMC12205521; doi:10.1186/s40478-025-02061-5)
Supplement: Supplementary file 1 — Supplementary Material 1 [file 40478_2025_2061_MOESM1_ESM.pdf]

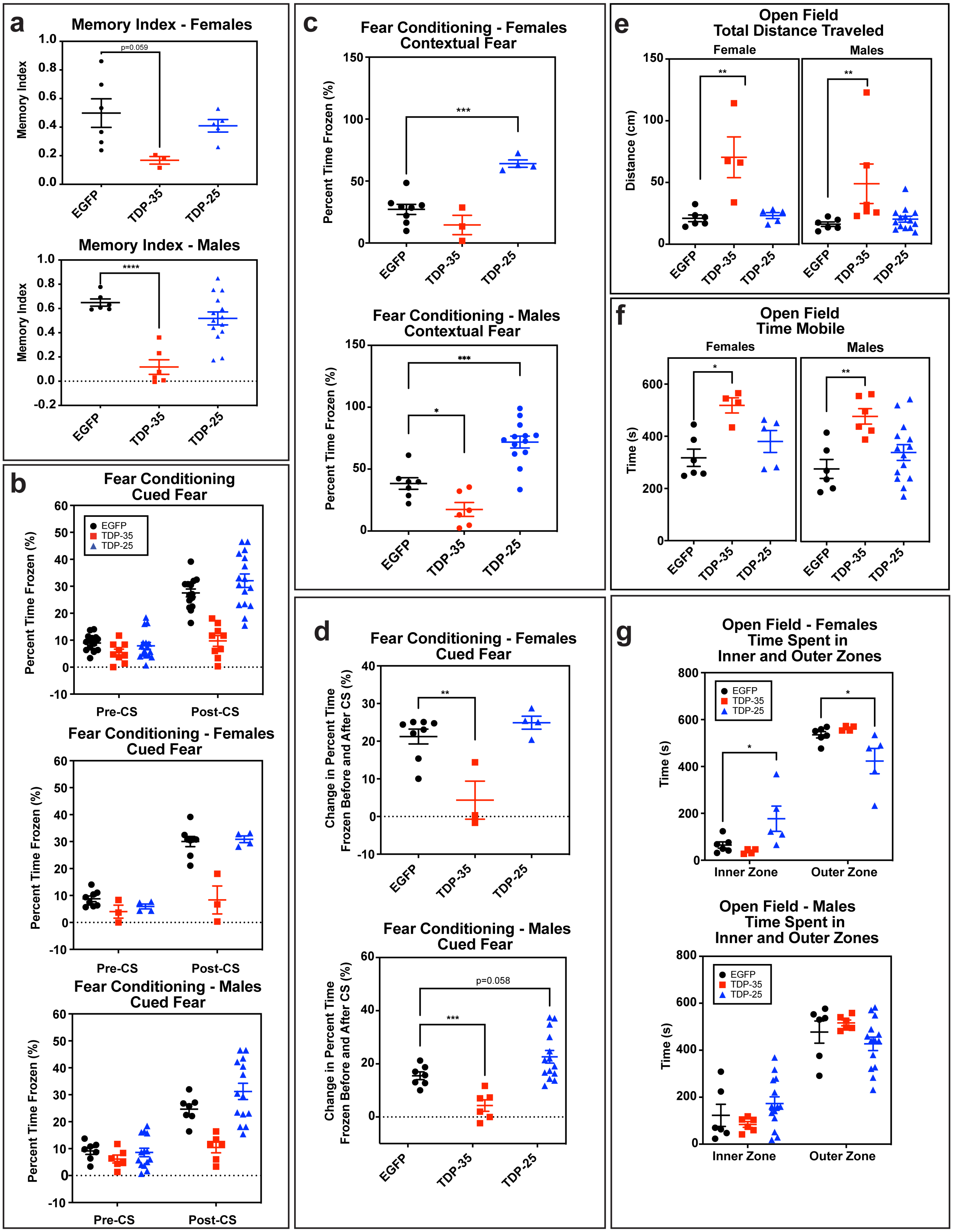

Supplementary Fig. 1 rAAV9-mediated expression of TDP-35 and TDP-25 result in distinct behavioral deficits, sex separated at 18 months. **a** Novel object recognition task. **b-d** Fear conditioning tasks. **e-g** Open field tasks. Significance values indicated using asterisks, where **** is P < .0001, *** is P < .001, ** is P < .01, and * is P< .05. At 6 months, n= 26F/24M (EGFP), 17F/23M (TDP-35), 25F/45M (TDP-25); at 12 months, n= 16F/9M (EGFP), 12F/12M (TDP-35), 10F/20M (TDP-25); at 18 months, n= 6F/6M (EGFP), 4F/6M (TDP-35), 4F/14M (TDP-25). Error bars represent standard errors of the mean, significance levels were calculated using Student's t test and Kruskal Wallis test.

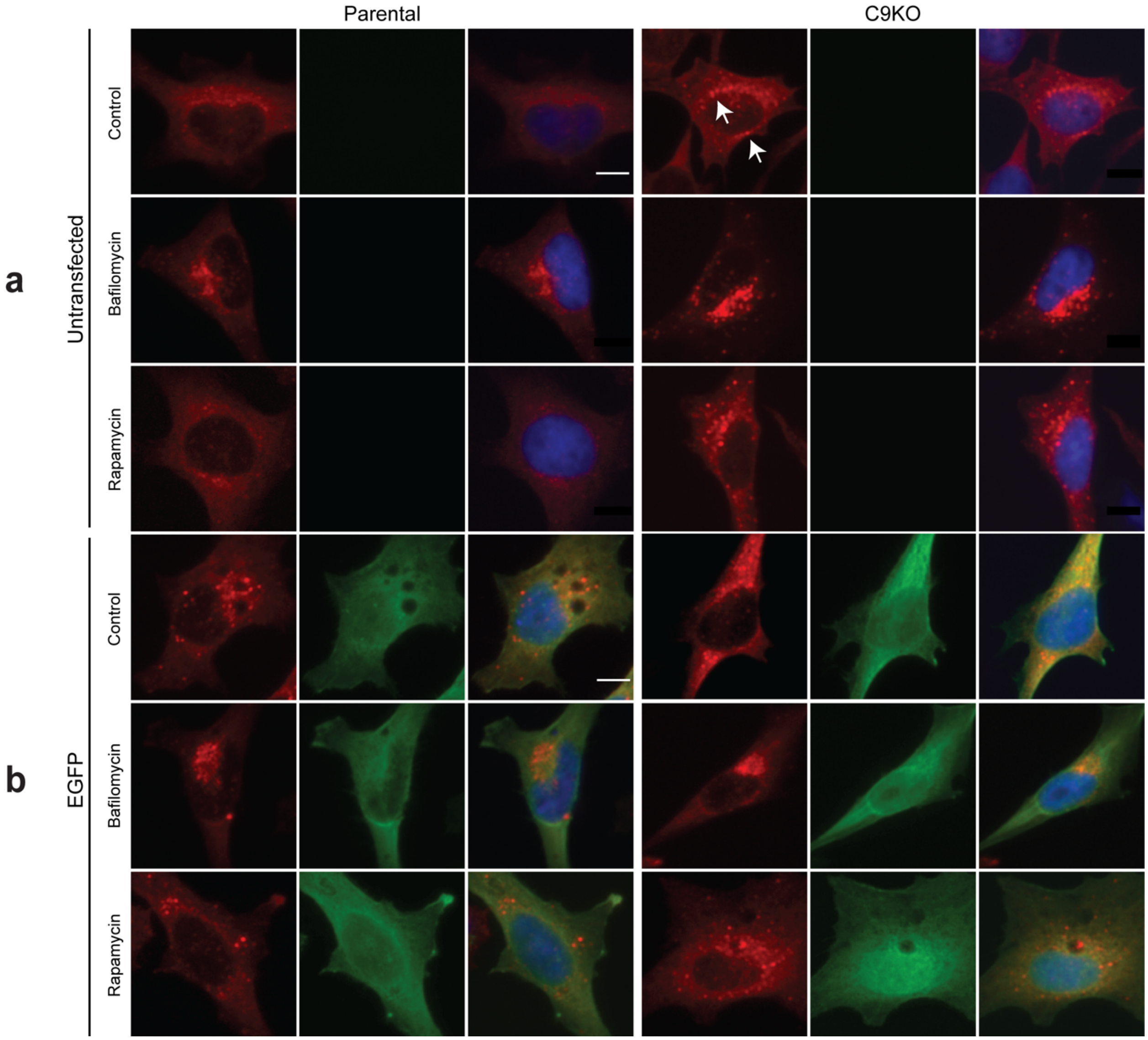

Supplementary Fig. 2 Autophagy modifiers affect p62 puncta formation in untransfected and EGFP-transfected HeLa cells. **a** Non-transfected and **b** EGFP transfected Parental C9KO HeLa cells treated with autophagy inhibitor, bafilomycin, and autophagy activator, rapamycin, stained for p62 (red), EGFP (green), and DAPI nuclear stain (blue). **a** Increased levels of cytoplasmic p62 puncta in non-transfected C9KO HeLa cells compared to parental controls, indicated with arrows. Bafilomycin treatment increased number of p62 puncta in both Parental and C9KO cells. Rapamycin had no observable effect on non-transfected parental cells or C9KO cells compared to untreated cells. **b** Similarly, C9KO cells expressing EGFP exhibited greater numbers of p62 puncta compared to the parental control, with increased p62 induced with bafilomycin and minor effects of rapamycin on reducing p62 puncta. Scale bar is 10 µm.

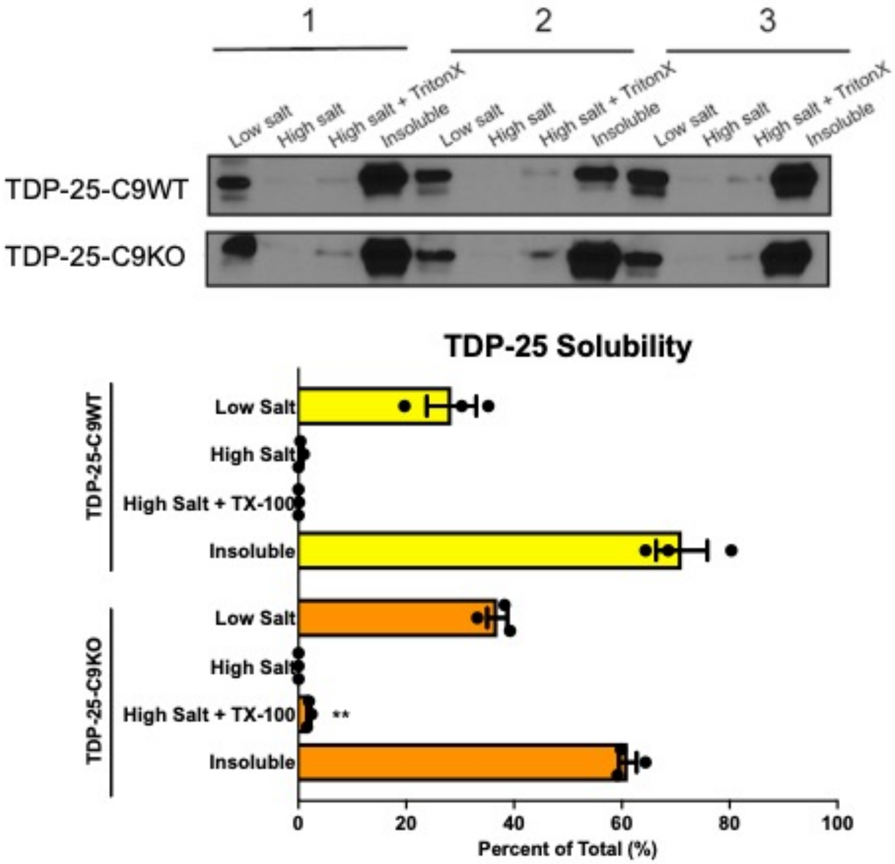

Supplementary Fig. 5 Altered TDP-25 Solubility was observed in C9orf72-deficient TDP-25 mice. All experiments were repeated with three biological replicates. Significance levels were calculated using Student's t-test. Significance values indicated using asterisks, where **** is P < .0001, *** is P < .001, ** is P < .01, and * is P < .05. Error bars represent standard errors of the mean.

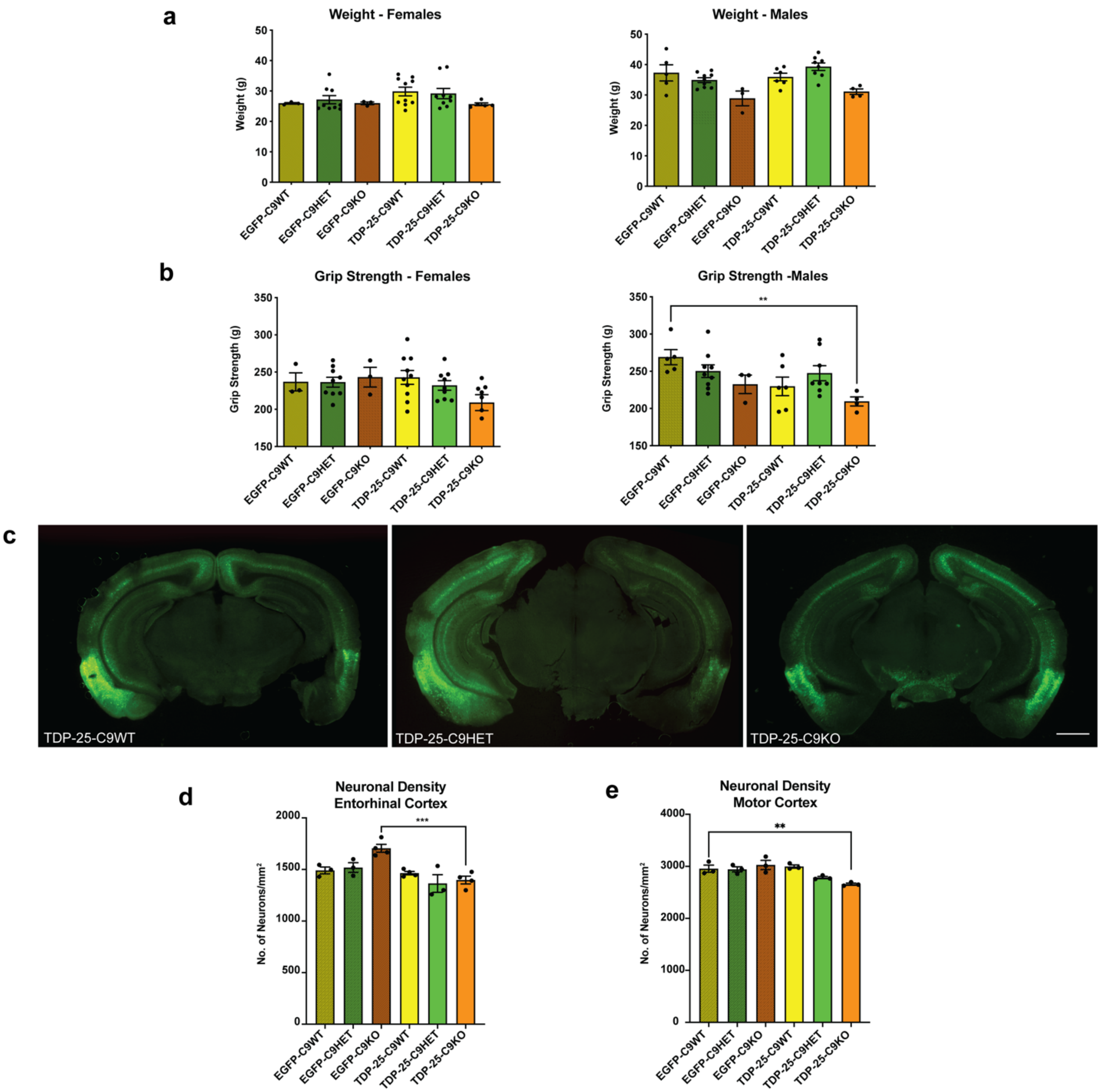

Supplementary Fig. 4 C9orf72 deficient TDP-43 proteinopathy mouse model at 8 months shows similar trends between male and female mice and no difference of expression pattern between WT, heterozygous, and homozygous C9KO mice. Sex separated results of **a** weight and **b** grip strength of 8-month-old mice. **c** representative images of the posterior hemisphere of TDP-25-C9WT, TDP-25-C9HET, and TDP-25-C9KO mice show similar TDP-25 expression patterns between the three genotypes. Green is EGFP. Scale bar is 1 mm. Neuronal density of the **d** entorhinal cortex and **e** motor cortex was quantified by the number of NeuN positive DAPI cells normalized to the area. n≥3, significance levels were calculated using Kruskal Wallis test and Student's t test. Significance values indicated using asterisks, where **** is P < .0001, *** is P < .001, ** is P < .01, and * is P < .05. Error bars represent standard errors of the mean.

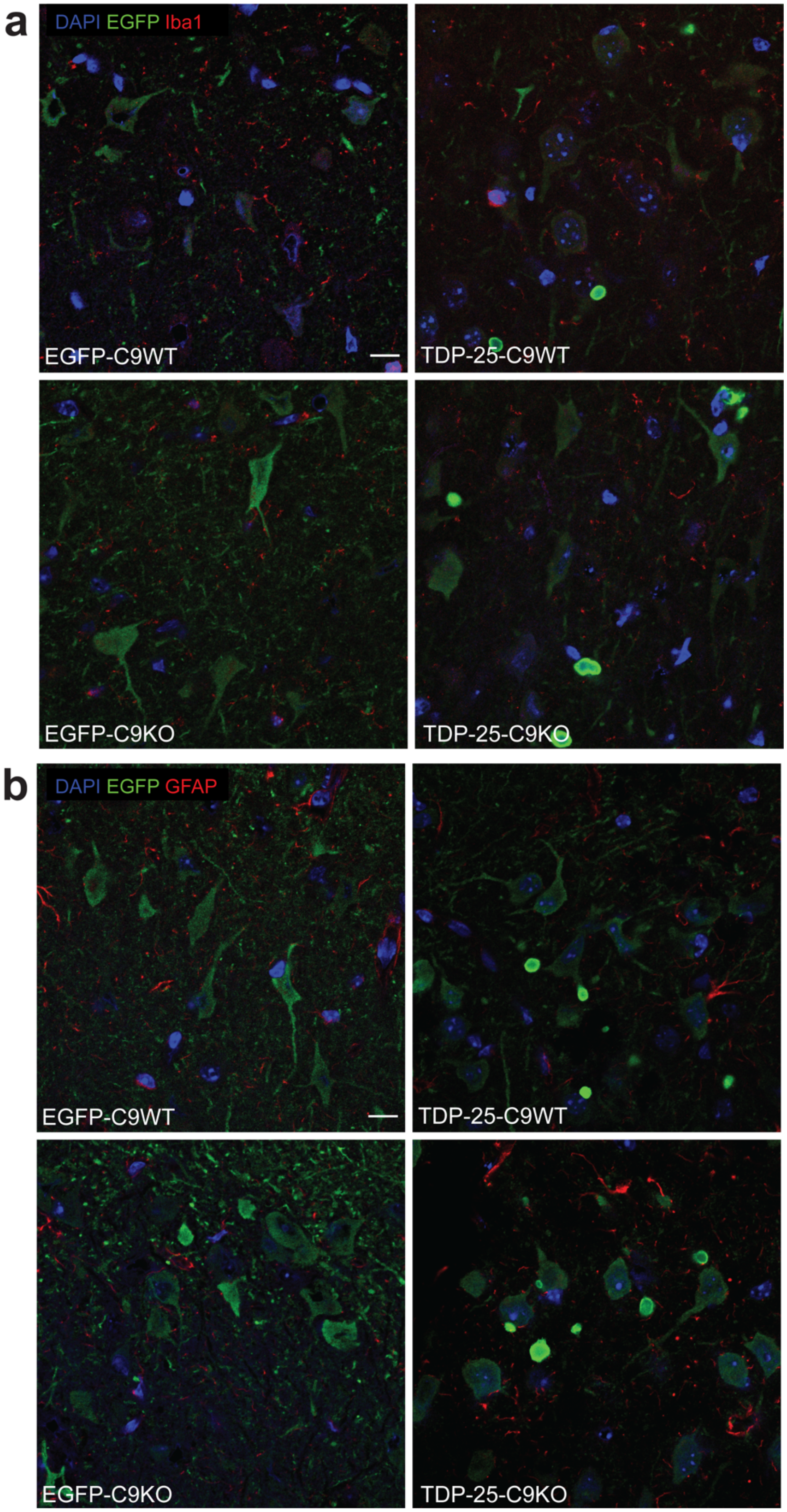

Supplementary Fig. 5 No overt neuroinflammation was observed in 8-month-old mice. Neuroinflammation was examined through **a** Iba1(red), a microglial marker, and **b** GFAP (red), a astrocytic marker, immunostaining and co-labeled with EGFP (green) and DAPI (blue). Scale bar is 10 μm. All experiments were repeated with three biological replicates.
