# Supplementary figures and images for: C9orf72 deficiency impairs the autophagic response to aggregated TDP-25 and exacerbates TDP-25-mediated neurodegeneration in vivo
Source: Acta Neuropathol Commun. 2025 Jun 28;13:136. doi: 10.1186/s40478-025-02061-5 (PMC12205521; doi:10.1186/s40478-025-02061-5)

Original Western Blots  
Figure 8:

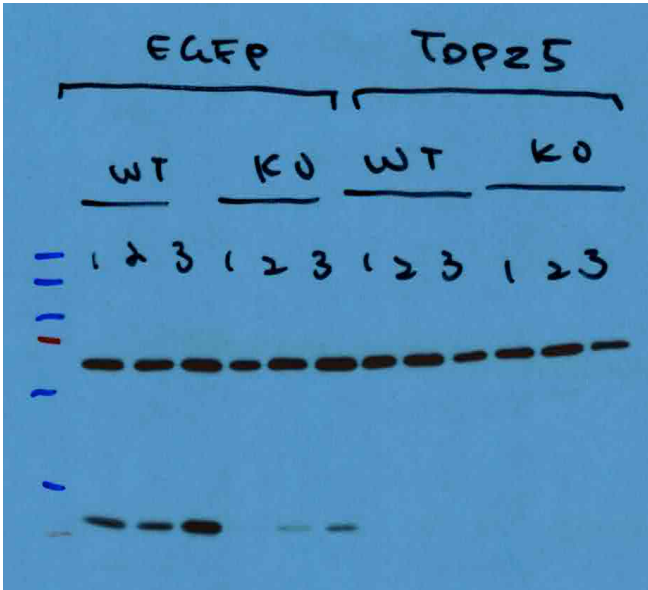

p62

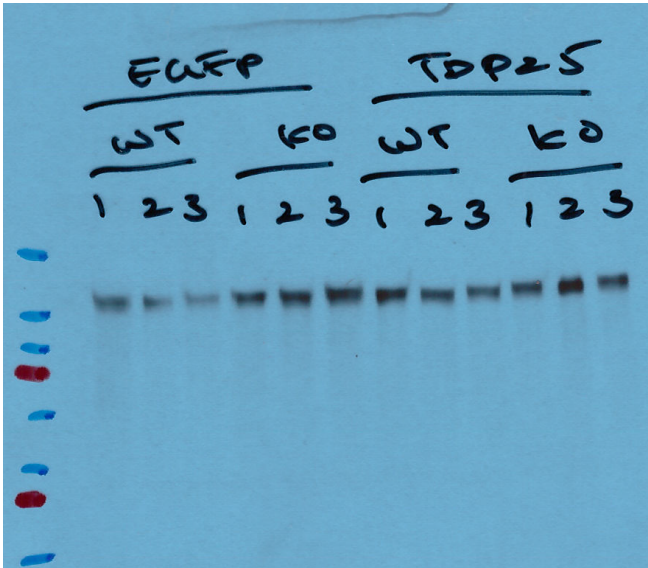

ULK1

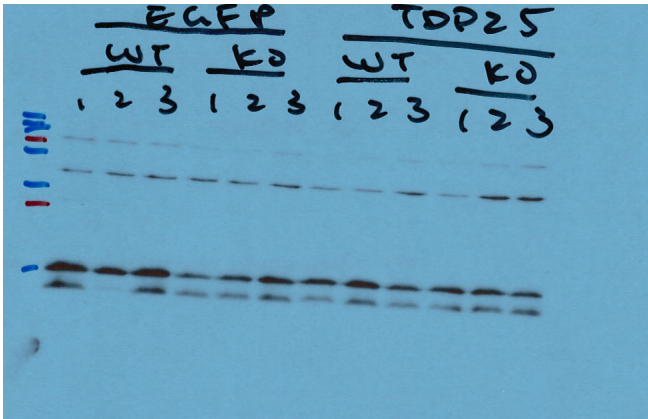

LC3A/B

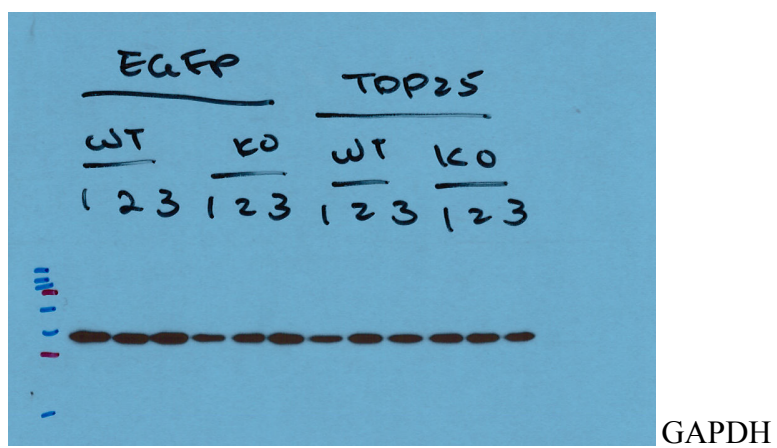

Supplementary Figure 3:

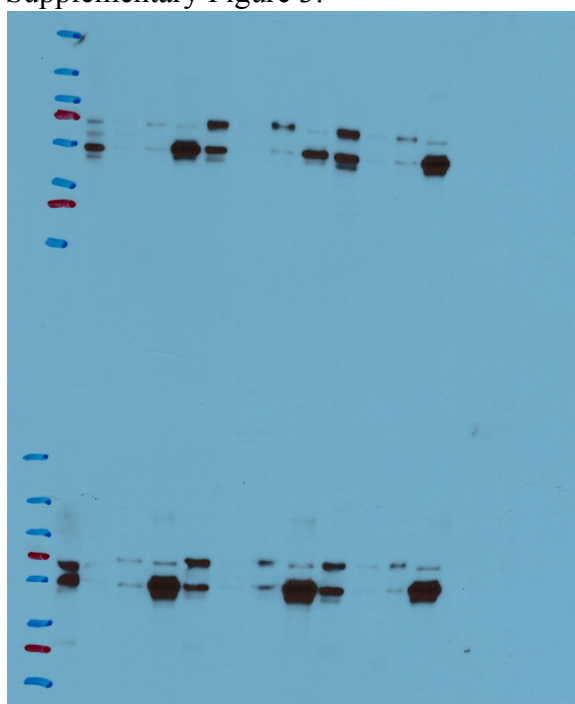

Supplement: Supplementary file 2 — Supplementary Material 2 [file 40478_2025_2061_MOESM2_ESM.pdf]
